# Eyes shut homolog is important for the maintenance of photoreceptor morphology and visual function in zebrafish

**DOI:** 10.1371/journal.pone.0200789

**Published:** 2018-07-27

**Authors:** Muriël Messchaert, Margo Dona, Sanne Broekman, Theo A. Peters, Julio C. Corral-Serrano, Ralph W. N. Slijkerman, Erwin van Wijk, Rob W. J. Collin

**Affiliations:** 1 Department of Human Genetics, Radboud University Medical Center, Nijmegen, The Netherlands; 2 Donders Institute for Brain, Cognition and Behaviour, Radboud University Medical Center, Nijmegen, The Netherlands; 3 Department of Otorhinolaryngology, Radboud University Medical Center, Nijmegen, The Netherlands; 4 Radboud Institute for Molecular Life Sciences, Radboud University Medical Center, Nijmegen, The Netherlands; University Zürich, SWITZERLAND

## Abstract

Mutations in *eyes shut homolog* (*EYS*), a gene predominantly expressed in the photoreceptor cells of the retina, are among the most frequent causes of autosomal recessive (ar) retinitis pigmentosa (RP), a progressive retinal disorder. Due to the absence of *EYS* in several rodent species and its retina-specific expression, still little is known about the exact function of EYS and the pathogenic mechanism underlying *EYS*-associated RP. We characterized *eys* in zebrafish, by RT-PCR analysis on zebrafish eye-derived RNA, which led to the identification of a 8,715 nucleotide coding sequence that is divided over 46 exons. The transcript is predicted to encode a 2,905-aa protein that contains 39 EGF-like domains and five laminin A G-like domains, which overall shows 33% identity with human EYS. To study the function of EYS, we generated a stable *eys*^*rmc101/rmc101*^ mutant zebrafish model using CRISPR/Cas9 technology. The introduced lesion is predicted to result in premature termination of protein synthesis and lead to loss of Eys function. Immunohistochemistry on retinal sections revealed that Eys localizes at the region of the connecting cilium and that both rhodopsin and cone transducin are mislocalized in the absence of Eys. Electroretinogram recordings showed diminished b-wave amplitudes in *eys*^*rmc101/rmc101*^ zebrafish (5 dpf) compared to age- and strain-matched wild-type larvae. In addition, decreased locomotor activity in response to light stimuli was observed in *eys* mutant larvae. Altogether, our study shows that absence of Eys leads to a disorganized retinal architecture and causes visual dysfunction in zebrafish.

## Introduction

Retinitis pigmentosa (RP) is a clinically diverse and genetically heterogeneous disorder with a prevalence of approximately 1 in 4,000 individuals. Retinitis pigmentosa is characterized by night blindness and visual field constriction, and often leads to total blindness [1]. For RP, different modes of inheritance including autosomal recessive, autosomal dominant, and X-linked, have been observed. To date, 58 genes are described in which mutations can cause arRP (RetNet, http://www.sph.uth.tmc.edu/RetNet/), most of them only being responsible for 1–2% of all arRP cases. Mutations in the *eyes shut homolog* (*EYS*) gene are among the most frequent causes of arRP, accounting for approximately 5–10% of all cases [2–4]. *EYS* is located on chromosome 6p12 and is predominantly expressed in the retina. Human *EYS* encodes for the 3,144 amino acid protein eyes shut homolog (EYS) that consists of five laminin A G-like (LamG) domains and 27 epidermal growth factor (EGF) or EGF-like domains [5, 6].

Eyes shut homolog is an ortholog of the *Drosophila* spacemaker (spam) protein, which plays a major role in the development of photoreceptors and the maintenance of the photoreceptor morphology [7]. Since the composition of the *Drosophila* eye is substantially different compared to that of the human eye, it remains questionable whether *Drosophila* can serve as a good model to study *EYS*-associated RP. Extensive bio-informatic analysis showed that *Eys* is absent from several rodent genomes (e.g. mice) [6], which also excludes these animals for being used as a model system to study Eys function. In the zebrafish genome, several gene predictions encoding EGF-like domains and LamG domains are predicted on chromosome 13, including Chr13.1401, Chr13.1402, Chr13.1403, ENSDART00000108504 and ENSDART00000122834. This indicates that *eys* probably is present in zebrafish, although its complete sequence was not characterized. Furthermore, the zebrafish retina is morphologically similar to that of the human retina, in the sense that all the major cell layers found in humans are also present in zebrafish [8], thereby providing a promising model to study the function of EYS.

Recently, two independent groups reported degeneration of the photoreceptor cell layer in different *eys* knock-out zebrafish models [9, 10]. Yu *et al*. generated an *eys* mutant zebrafish line using CRISPR/Cas9 technology in which degradation of the outer nuclear layer was observed. In addition, they revealed that eys localizes near the photoreceptor connecting cilium and that it might play a role in maintaining the ciliary pocket [10]. In a study of Lu *et al*., TALEN technology was used to generate an *eys* knock-out zebrafish, which showed visual impairment by recording electroretinography (ERG). Furthermore, they demonstrated F-actin disruption and mislocalization of the retinal proteins red opsin, UV opsin and rhodopsin in the absence of Eys [9]. Both studies imply a role of Eys in maintaining retinal morphology and visual function, although the exact mechanism underlying *EYS*-associated RP still remains to be elucidated.

In this study, we generated a stable *eys*^*-/-*^ mutant zebrafish line, designated *eys*^*rmc101/rmc101*^, using CRISPR/Cas9 technology to study the function of Eys in the zebrafish retina. Similar to previous studies [9, 10], we observed progressive degeneration of the photoreceptor outer segments, mislocalization of rhodopsin and decreased ERG responses in absence of Eys. In addition, we show diminished locomotor activity of *eys*^*rmc101/rmc101*^ zebrafish larvae in response to light stimuli, which has not been shown before.

## Materials and methods

### Animal experiments

The zebrafish experiments were approved by the Centrale Commissie Dierproeven (CCD, RU-DEC 2016–0091) and were performed in accordance with the Dutch law (Wet op de Dierproeven 1996) and European regulations (Directive 86/609/EEC).

### Fish husbandry

We used the in-house bred Tupfel Longfin strain. Adult zebrafish were maintained in 4.5-liter polyethylene tanks (Tecniplast) in an Aqua Schwarz holding system (Göttingen) supplied continuously with circulating filtered tap water (electoral conductivity of 300 uS cm-1, 27.5°C, pH 7.5–8) under cycles providing 14 hrs of light and 10 hrs of dark (14:10 LD; lights on 9 AM; lights off 11 PM). The fish were fed with Gemma Micro 300 (Skretting) twice a day and living Artemia once a day.

### RNA isolation

Total RNA from adult zebrafish eyes was obtained by snap-freezing isolated eyes in liquid nitrogen and subsequently homogenizing the eyes in Trizol (Thermo Fischer Scientific) using a pestle and moving through a syringe five times. After incubation on ice for 15 minutes, 250 μl chloroform was added, incubated for 3 minutes and centrifuged at 4°C at 12,000xg for 15 minutes. The supernatant was mixed with 1 μl glycogen (5 μg/μl) and 250 μl isopropanol and stored at -80°C o/n. The RNA was further purified and DNAse treated using RNeasy spin columns (Qiagen) according to manufacturer’s protocol.

### RT-PCR analysis of *eys*

For cDNA synthesis, one μg of total RNA was reverse transcribed using the iScript^™^ cDNA synthesis kit (Bio-Rad) according to the manufacturer’s instructions. For the characterization of zebrafish *eys*, RT-PCR was performed. One microliter cDNA was incubated together with 0.5 μM of the forward and reverse primer, 100 μM dNTPs (Roche), 0.25 U Taq polymerase (Roche), 10x PCR buffer + 15 mM MgCl_2_ (Roche) and 1x Q solution (Qiagen) in a total volume of 25 μl. The following program was run in a 2720 Thermal Cycler (Applied Biosystems): the samples were denatured at 94°C for 3 minutes followed by 35 cycles of amplification consisting of 94°C for 20 seconds, 58°C for 30 seconds and 72°C for 50 seconds, and a final primer extension at 72°C for 5 minutes. Primers targeting all exons of *eys* were designed, based on gene predictions Chr13.1401, Chr13.1402, Chr13.1403, ENSDART00000108504 and ENSDART00000122834 using the UCSC Genome Browser on Zv9/danRer7 assembly. Primer sequences are listed in S1 Table. A number of these primer combinations were used for nested PCRs resulting in the amplification of PCR products representing parts of the *eys* transcript expressed in zebrafish eyes. To verify that the amplified products indeed correspond to *eys*, PCR products were purified on Nucleospin Gel and PCR Clean-up columns (Macherey Nagel) and either directly sequenced or cloned in the pCR4-TOPO vector with the use of the TOPO TA Cloning Kit (Invitrogen) for sequencing with T7 and T3 sequencing primers.

To study the presence of *eys* transcript in wild-type and *eys*^*rmc101/rmc101*^ larvae, total RNA from 20 pooled larvae was extracted as described above. RT-PCR was performed using a primer pair covering the region in exon 20 of *eys* where the deletion was located (S1 Table).

### Multiple sequence alignment

A multiple sequence alignment of EYS proteins from different species was generated using AlignX in the Vector NTI software package (Vector NTI Advance 11). Accession numbers of the protein sequences used for sequence comparison are as follows: human, NM_001142800.1 (RefSeq); macaque, XM_011737495.1 (RefSeq); chicken, XM_015284845.1 (RefSeq); *Drosophila*, NM_001032399.3 (RefSeq).

### Target site selection and gRNA synthesis

Target sites for genome editing were selected by using the online web tool CHOPCHOP (https://chopchop.rc.fas.harvard.edu/) [11]. Guide RNAs (gRNAs) were synthesized as described previously [12]. Templates for gRNA transcription were generated by annealing gene-specific oligonucleotides containing the T7 (5’-TAATACGACTCACTATA-3’) promoter sequence, the 20-base target sequence without the PAM, and a complementary region to a constant oligonucleotide encoding the reverse complement of the tracrRNA tail. T4 DNA polymerase (New England Biolabs) was used to fill the ssDNA overhang and the template was then purified using the GenElute PCR clean-up kit (Sigma). Transcription of the gRNAs was performed using the T7 MEGAshortscript Kit (Ambion). Oligos used for gRNA synthesis are listed in S2 Table.

### Microinjections

Zebrafish embryos were collected after natural spawning and injected at the single cell stage with one nanoliter of injection mix (4.5 μl gRNA (1μg/μl), 2.5 μl Cas9 protein (2μg/μl, PNA Bio), 2 μl 1M KCl and 1 μl 0.5% phenol red dye). To screen for genomic lesions, genomic DNA was extracted from a pool of 15 embryos at 2.5 days post fertilization (dpf).

### Genotyping

Genomic DNA was extracted from larvae at 2.5 dpf or caudal fin tissue from adult zebrafish. Tissue was incubated in 25 μl (larvae) or 75 μl (fin tissue) lysis buffer (40 mM NaOH 0.2 mM EDTA) at 95°C for 20 minutes. The lysed samples were diluted 10 times of which 1 μl was incubated together with 0.5 μM of the forward and reverse primer, 100 μM dNTPs (Roche), 0.25 U Taq polymerase (Roche) and 10x PCR buffer + 15 mM MgCl_2_ (Roche) in a total volume of 25 μl. Samples were denatured at 94°C for 3 minutes followed by 35 cycles of amplification consisting of 20 seconds at 94°C, 30 seconds at 58°C and 50 seconds at 72°C, and a final primer extension of 5 minutes at 72°C. To screen for genomic lesions, PCR products were sequenced directly. Primer sequences are listed in S1 Table.

### Immunohistochemistry

Dissected adult zebrafish eyes (2 mpf and/or 5 mpf) and larvae (5 dpf) from *eys*^*rmc101/rmc101*^ mutants and their age- and strain-matched wild-type controls were cryoprotected with 10% sucrose in PBS for 10 minutes prior to embedding in OCT compound. Embedded samples were snap frozen in liquid nitrogen-cooled isopentane and cryosectioned following standard protocols. Cryosections (7 μm) were fixed with 4% paraformaldehyde (PFA) at room temperature for 10 minutes, permeabilized with 0.01% PBS-Tween20 and hereafter blocked in 10% normal goat serum and 2% BSA in PBS at room temperature for 1 hour. Subsequently, they were incubated with primary antibodies directed against EYS/RP25 (rabbit, 1:300, Novus Biological NBP1-90038), Centrin (mouse, 1:250, Millipore 04–1624) or GFAP (rabbit, 1:750, Dako) at 4°C overnight. After washing with PBS, cryosections were incubated with secondary antibodies (rabbit IgG, Alexa Fluor 488, goat, 1:800, Molecular Probes A11008; mouse IgG, Alexa Fluor 568, goat, 1:800, Life Technologies A11031) at room temperature for one hour.

For rhodopsin and cone transducin staining, zebrafish eyes were fixed with 4% PFA at 4°C overnight, followed by incubation in a MeOH gradient. After embedding, freezing and cryosectioning, slides were permeabilized with 0.1% PBS-Tween20 and incubated in antigen retrieval solution (10 mM sodium citrate pH 8.5) at 121°C for 1 minute. After washing, cryosections were blocked in block solution (10% Non Fat Dry Milk in 0.1% PBS-Tween20) at RT for 1 hour, followed by incubation with the primary antibodies Rhodopsin (clone 4D2, mouse, 1:2,000, Novus Biological NBP1-48334) and GNAT2 (rabbit, 1:500, MBL PM075) in block solution at 4°C overnight. After washing with 0.1% PBS-Tween20, slides were incubated with the secondary antibodies (rabbit IgG, Alexa Fluor 488, goat, 1:800, Molecular Probes A11008; mouse IgG, Alexa Fluor 488, goat, 1:800, Invitrogen A11029) together with Phalloidin Alexa Fluor 568 (1:100, Molecular Probes, A-12380).

For staining with boron-dipyrromethene (BODIPY), zebrafish larvae or eyes were fixed in 4% PFA for 2 hours, cryoprotected in 10% sucrose in PBS for 30 minutes, embedded in OCT, snap frozen in melting isopentane and cryosectioned. BODIPY was applied to the cryosections at room temperature for 20 minutes.

In all cases, nuclei were counterstained with DAPI (1:8,000). Imaging was performed on a ZEISS LSM 880 microscope with Airyscan. Zen software was used for processing the images. To analyze the thickness of ONL and INL in 5 dpf, 2 mpf and 5 mpf zebrafish, up to 10 different measurements were taken for each section. Two different sections were analyzed for one wild-type fish and one *eys* mutant fish per time-point.

### Optokinetic response (OKR) measurements

Optokinetic response measurements were performed as described previously [13]. In brief, zebrafish larvae were mounted in 3% methylcellulose in a small Petri-dish, which was placed on a platform surrounded by a rotating drum of 8 cm in diameter. A pattern of alternating black and white vertical stripes was displayed on the drum interior (each stripe subtended an angle of 36°C). Larvae (5 dpf) were placed in an upright position and visualized through a stereomicroscope positioned over the drum and illuminated with fiberoptic lights. Eye movements were recorded while larvae were optically stimulated by the rotating stripes. Larvae were subjected to a protocol of 30 seconds 5 rpm counterclockwise rotation, 10 seconds rest, 30 seconds 5 rpm clockwise rotation, 10 seconds rest, 30 seconds 8 rpm counterclockwise rotation, 10 seconds rest, 30 seconds 8 rpm clockwise rotation. The amount of eye movements were counted from the recorded movies afterwards and plotted using Graphpad Prism (version 5.03).

### Electroretinography (ERG)

Electroretinography measurements were performed on isolated larval eyes (5 dpf) as previously described [14]. Larvae were dark-adapted for a minimum of 30 minutes prior to the measurements, and subsequently handled under dim red illumination. The isolated eye was positioned to face the light source. Under visual control via a standard microscope (Stemi SV8, Zeiss) equipped with red illumination (KL1500 electronic, Zeiss), the recording electrode with an opening of approximately 15 μm at the tip was placed against the center of the cornea with a micromanipulator. This electrode was filled with E3 medium (5 mM NaCl, 0.17 mM KCl, 0.33 mM CaCl, and 0.33 mM MgSO4). A custom-made stimulator was invoked to provide light pulses of 100 ms duration, with a 100% light intensity of 6000 lux. It uses a ZEISS XBO 75W light source and a fast shutter (custom made) driven by the self-developed NI Labview program. Electronic signals were amplified 1,000 times by a pre-amplifier (P55 A.C. Preamplifier, Astro-Med. Inc, Grass Technology) with a band pass between 0.1 and 100 Hz, digitized by DAQ Board NI BNC-2090 (National Instruments) and displayed via the self-developed NI Labview program. All experiments were performed at room temperature.

### Visual motor response

Locomotor activity in response to light-dark conditions, also known as visual motor response (VMR), was analyzed using the Danio Vision system (Noldus B.V.). Collected embryos were raised in E3 medium (5mM NaCL, 0.17mM KCL, 0.33mM CaCl_2_, 0.33mM MgSO_4_, supplemented with 0.1% methylene blue) in a 28°C incubator, subsequently under 14 hrs of light and 10 hrs of dark. Medium was changed every day and during the process curved and dead larvae were discarded. Behavioral tests were carried out at 5 dpf. Larvae were transferred to a 48-wells plate filled with 200 μl E3 medium without methylene blue. In each run, 24 mutant larvae and 24 age- and strain-matched control wild-type zebrafish larvae were tested. During the experiment, the temperature was kept constant at 28°C using a heating/cooling system (Noldus B.V.). The protocol consisted of 20 min acclimation (with lid of the system open; room light: 500–650 lux), closing of the lid followed by alternating periods of 10 minutes dark, 10 minutes bright light (about 3000 lux) and 10 minutes dark (in total 12 cycles). Variables of interest were: *distance moved* (mm) and *maximum velocity* (Vmax, mm/s), and *difference in distance moved* and *difference in Vmax* (maximum distance moved/Vmax of first 30 seconds light condition minus average distance moved/Vmax last 30 seconds of dark condition) for the change from dark to light.

### Statistical analysis

Statistical analysis was performed using Graphpad Prism software. For data obtained using Danio Vision, RStudio version 1.0.153 (https://www.r-project.org) was used to generate graphs and perform statistical analysis. The difference between wild-type and mutant zebrafish was analyzed using two-tailed, unpaired Student’s *t*-test, and *p*-values were corrected for multiple testing using the Benjamini-Hochberg method. Statistical significance was set at *p*< 0.05. The means and standard errors of the mean are shown. Exact *p*-values are shown in S3 Table.

## Results

### Characterization of zebrafish *eys* cDNA

The complete zebrafish *eys* sequence was not completely identified, however, in the UCSC genome browser, several gene predictions for zebrafish *eys* were present. On zebrafish chromosome 13, genes encoding EGF-like domains and LamG domains were predicted, including Chr13.1401, Chr13.1402, Chr13.1403, ENSDART00000108504 and ENSDART00000122834 (S1 Fig). Based on these gene predictions, we hypothesized that *eys* is present in zebrafish. We performed RT-PCR using primers targeting *eys* on RNA derived from adult zebrafish eyes as a template. This resulted in the identification of a 8,715 nucleotide long transcript encompassing 46 exons. Most of these exons were previously predicted by several gene prediction programs, although also a number of new exons were identified (S1A Fig). The resulting *eys* transcript translates into a protein of 2,905 amino acids that is predicted to harbor 39 EGF-like domains and five LamG domains. This domain organization is similar to what is observed for the human and *Drosophila* EYS proteins (S1B Fig). Interestingly, the zebrafish Eys protein appears to lack a so called low-complexity region, as seen for the human and *Drosophila* protein, in which no domains are predicted. In human, this region is completely encoded by exon 26, which is lacking in zebrafish *eys*.

We performed a multiple sequence alignment of the predicted Eys protein with the sequence of Eys from several other species (S2 Fig). From this, we could deduce that the amino acids coding for the N-terminal EGF- and EGF-like domains as well as the C-terminal LamG domains are conserved over macaque, chicken, *Drosophila*, human and zebrafish. Overall, the predicted zebrafish Eys protein shows a 33% sequence identity compared to human EYS.

### Generation of an *eys*^*-/-*^ zebrafish line by CRISPR/Cas9 technology

**Fig 1:**
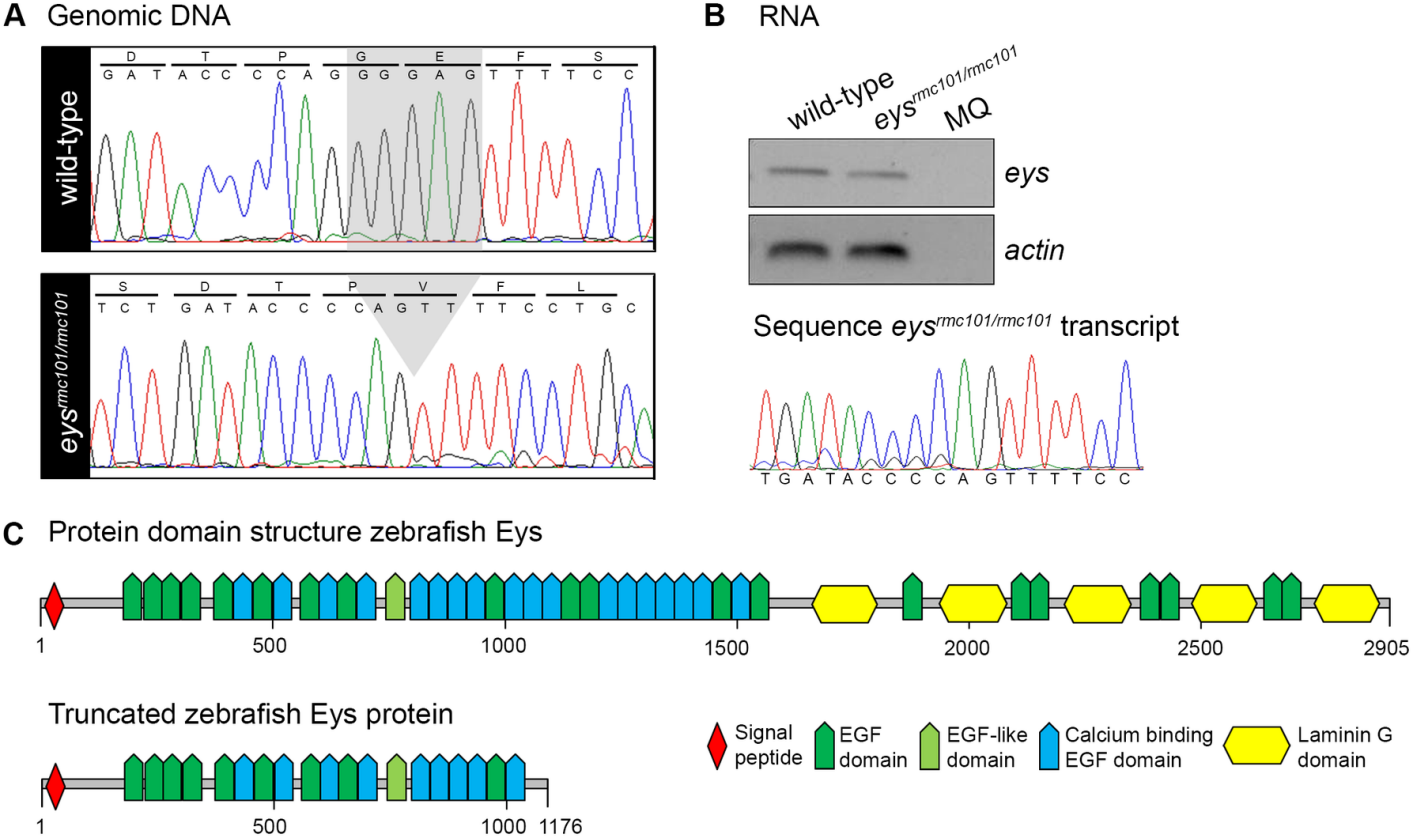
Characterization of a stable *eys*^*rmc101/rmc101*^ zebrafish line. **(A)** Sanger sequencing identified a five base pair deletion in exon 20 in *eys*^*rmc101/rmc101*^ zebrafish. **(B)** Representative gel image of RT-PCR analysis using RNA from a pool of larvae (n = 15), which shows that *eys* transcripts are present in both wild-type and *eys*^*rmc101/rmc101*^ zebrafish (upper panel). Sanger sequencing confirmed the presence of the five base pair deletion in the *eys*^*rmc101/rmc101*^ transcript (lower panel). **(C)** Protein domain structures of wild-type Eys and the truncated Eys protein that is predicted in *eys*^*rmc101/rmc101*^ zebrafish.

To better study the zebrafish Eys protein in the retina, we generated a stable zebrafish *eys* knock-out line using CRISPR/Cas9 technology. By screening zebrafish larvae for genetic lesions, several different types of mutations were identified. For subsequent experiments, zebrafish carrying a five base pair deletion (c.3488_3492del) in exon 20 were selected for the creation of a stable zebrafish line and named *eys*^*rmc101/rmc101*^(Fig 1A). The deletion is predicted to lead to a frameshift and premature termination of protein translation (p.Gly1163Valfs*14) (Fig 1C). To determine whether *eys* mRNA is present in homozygous mutant zebrafish or wether this mRNA undergoes nonsense mediated decay, RNA was isolated from a pool of larvae (n = 15). Subsequently, the presence of *eys* transcripts was determined by RT-PCR analysis. PCR products were observed for both wild-type and mutant zebrafish larvae (Fig 1B), indicating that mutant *eys* transcripts are not rapidly degraded. Sanger sequencing confirmed that *eys* knock-out fish express the *eys* mRNA with the five base pair deletion.

### Eys is absent from the retina of *eys*^*rmc101/rmc101*^ zebrafish and localizes with the ciliary protein centrin

**Fig 2:**
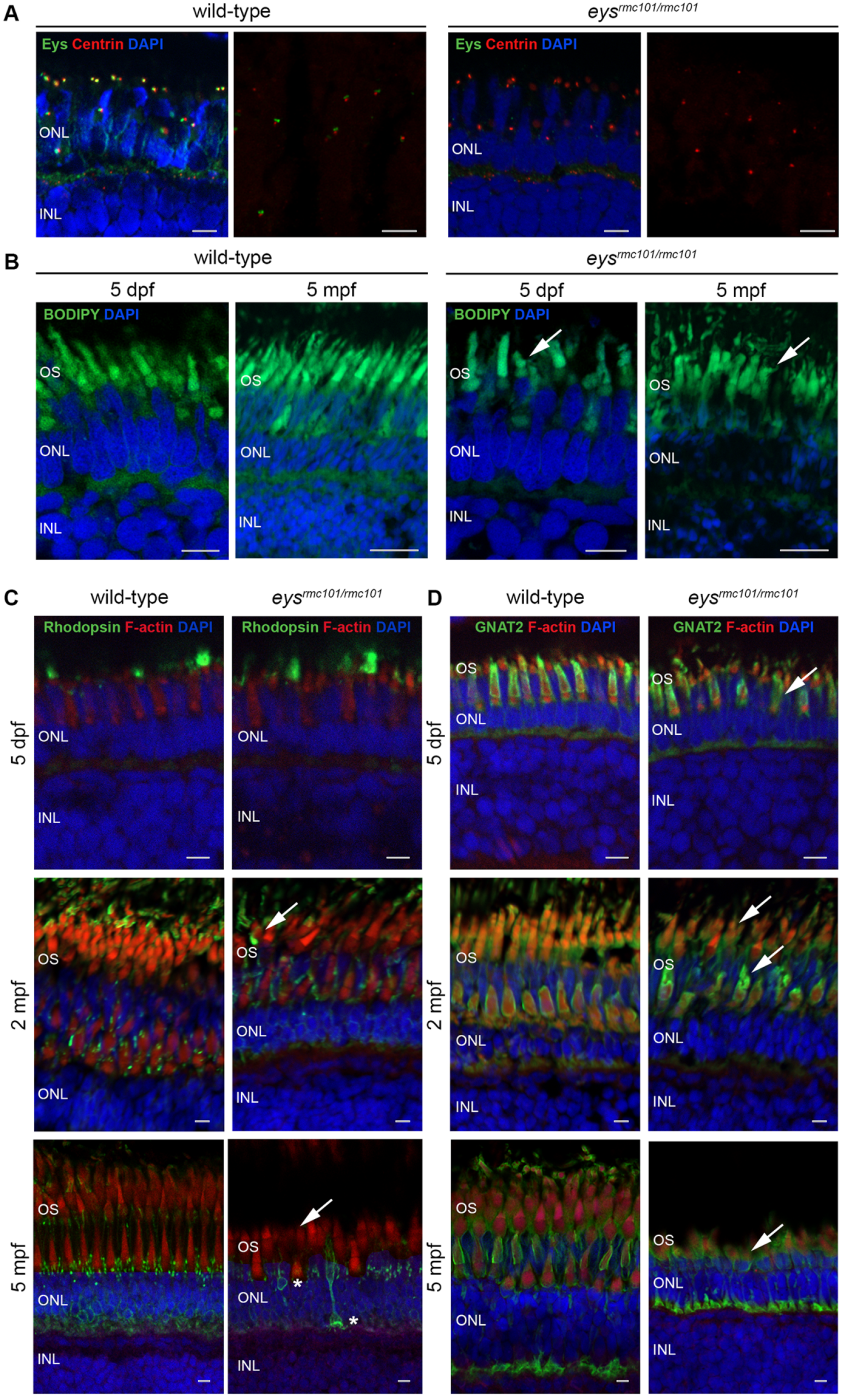
Immunohistochemistry on retinal sections of wild-type and *eys*^*rmc101/rmc101*^ zebrafish. **(A)** Retinal sections of wild-type and *eys*^*rmc101/rmc101*^ zebrafish at 5 dpf and 5 mpf stained with antibodies against Eys (green) and centrin (red). **(B)** BODIPY (green) staining showing disorganization of photoreceptor outer segments in *eys*^*rmc101/rmc101*^ zebrafish (5 dpf and 5 mpf) compared to age- and strain-matched wild-type zebrafish (arrows). **(C)** Retinal sections of wild-type and *eys*^*rmc101/rmc101*^ zebrafish at 5 dpf (upper panel), 2 mpf (middle panel) and 5 mpf (lower panel) stained with antibodies against rhodopsin (green) and F-actin (red). Asterisks indicate mislocalization of rhodopsin to the inner segments and synapses of photoreceptor cells. **(D)** Retinal sections of wild-type and *eys*^*rmc101/rmc101*^ zebrafish at 5 dpf, 2 mpf and 5 mpf stained with antibodies against GNAT2 (green) and F-actin (red). Arrows indicate dysmorphic outer segments in mutant zebrafish. In all images, nuclei are counterstained with DAPI (blue). INL: inner nuclear layer; ONL: outer nuclear layer; OS: outer segments. Scale: 5 μm.

To determine the localization of the Eys protein in the zebrafish retina, immuno-histochemistry with an antibody against Eys was performed on retinal cryosections of zebrafish at 5 days post fertilization (dpf) and 5 months post fertilization (mpf). Co-staining of wild-type and mutant retinas using the Eys antibody together with an antibody against centrin, a marker for the connecting cilium, was performed to determine whether Eys localizes near the connecting cilium. Eys expression was observed adjacent to the immunoreactive signal of centrin (Fig 2A). This suggests that Eys localizes near the photoreceptor connecting cilium. Since two puncta of Eys were always observed close to the immunofluoresence signal of centrin, it might be that Eys is located in the ciliary basal body and the daughter centriole. The Eys signal was completely absent in the retinas of *eys* knock-out zebrafish, indicating that the Eys protein is not properly expressed in the mutants and that the immunofluorescence signal observed in the wild-type retinas is specific (Fig 2A).

### Disorganization of photoreceptor outer segments and mislocalization of outer segments proteins in *eys*^*rmc101/rmc101*^ zebrafish

Next, we evaluated the effect of Eys deficiency on overall photoreceptor outer segment morphology by staining with BODIPY. This is a fluorescent dye that stains lipids and allows to visualize the outer segments of the photoreceptor cells. In the mutant retinas, the outer segments appear to be less in numbers, shorter, thicker and also more disorganized compared to outer segments in the wild-type retinas (Fig 2B). In addition, a reduction in inner and/or outer nuclear thickness was observed in *eys*^*rmc101/rmc101*^ fish of 2 mpf and 5 mpf (S3 Fig). As Müller glia activation is a feature of retinal degeneration in zebrafish, we investigated the presence of activated Müller glia cells by immunohistochemistry. Müller glia cells were detected using an antibody against a specific cytoskeletal protein, glial fibrillary acidic protein (GFAP). Glial fibrillary acidic protein is described to be upregulated in glial cells in response to injury [15]. However, we did not observe any differences in GFAP staining between wild-type and *eys*^*rmc101/rmc101*^ zebrafish, at 5 dpf nor at 2 mpf (S4 Fig). In addition, we stained the fish retinas (5 dpf, 2 mpf and 5 mpf) with an antibody against F-actin. In contrast to what was observed in the wild-type retinas, F-actin expression was disrupted in retinas of *eys*^*rmc101/rmc101*^ zebrafish (Fig 2C and 2D), which further illustrates outer segment disorganization and is in agreement with findings by Lu et al. 2017 [9]. Finally, we examined the localization of the photoreceptor proteins rhodopsin and cone transducin (GNAT2) in retinas of wild-type and *eys*^*rmc101/rmc101*^ larvae and adult zebrafish (5 dpf, 2 mpf and 5 mpf). In the wild-type retinas, rhodopsin localization was observed in the outer segments as well as lining the outer segment membranes (Fig 2C). Cone transducin was mainly observed lining the outer segments (Fig 2D). In retinas of *eys*^*rmc101/rmc101*^ adult zebrafish and larvae, dysmorphic outer segments were observed together with aberrant localization of rhodopsin and cone transducin (Fig 2C and 2D). In the retinas of *eys* mutant fish at 5 mpf, rhodopsin was partially mislocalized to the inner segments and synapses of photoreceptor cells (Fig 2C). This suggests that the change in outer segment morphology due to absence of Eys also affects the proper localization of other photoreceptor-specific proteins.

### Visual function is impaired in *eys*^*rmc101/rmc101*^ zebrafish

**Fig 3:**
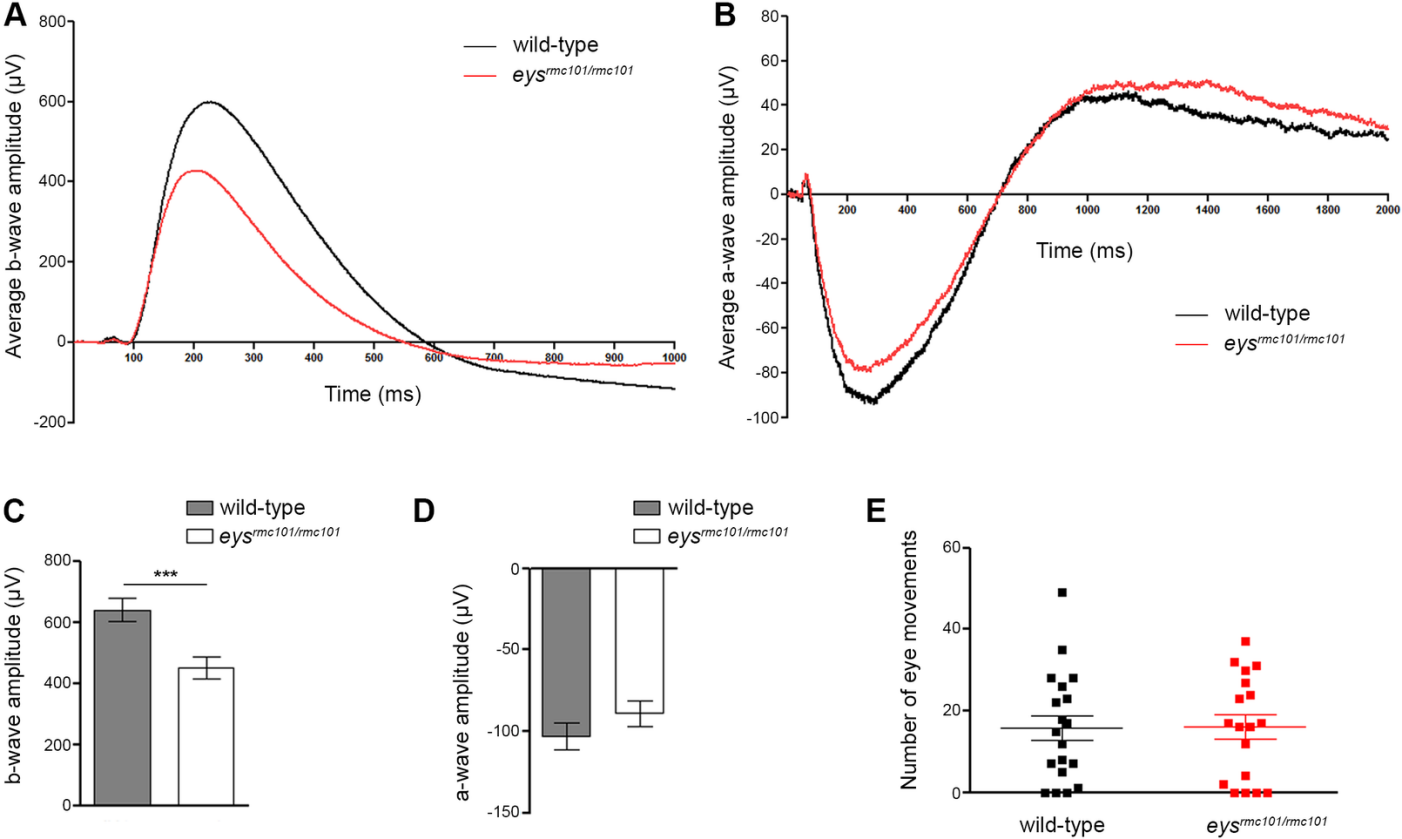
Visual function of wild-type and *eys*^*rmc101/rmc101*^ zebrafish larvae. **(A)** ERG measurements of the b-wave of wild-type (black line) and *eys*^*rmc101/rmc101*^ (red line) zebrafish larvae at 5 dpf. **(B)** ERG measurements of the a-wave of wild-type (black line) and *eys*^*rmc101/rmc101*^ (red line) zebrafish larvae at 5 dpf. **(C)** Quantification of the ERG b-wave amplitude of wild-type and *eys*^*rmc101/rmc101*^ zebrafish larvae at 5 dpf (n = 30; *p* = 0.0004). **(D)** Quantification of the ERG a-wave amplitude of wild-type and *eys*^*rmc101/rmc101*^ zebrafish larvae at 5 dpf (n = 20; *p* = 0.2324). **(E)** Optokinetic response measurements of wild-type and *eys*^*rmc101/rmc101*^ larvae at 5 dpf (n = 19).

To assess whether depletion of Eys results in visual dysfunction, we performed behavioral and functional assays. All assays were performed with larvae at 5 dpf. First, ERG recordings were performed to measure the visual function of wild-type and *eys*^*rmc101/rmc101*^ zebrafish larvae at 5 dpf. Mutant larvae showed significantly diminished b-wave amplitudes (640.4 μV ± 37.31 (mean ± SEM, n = 30)) compared to the b-wave amplitude of wild-type zebrafish larvae (451.0 μV ± 34.37 (mean ± SEM, n = 30)) (Fig 3A and 3C). No difference was observed in a-wave amplitude between wild-type (-102.9 μV ± 8.047 (mean ± SEM, n = 20)) and *eys*^*rmc101/rmc101*^ larvae (-89.06 μV ± 8.040 (mean ± SEM, n = 20)) (Fig 3B and 3D). The diminished b-wave amplitude in the mutant larvae indicates that the *eys*^*rmc101/rmc101*^ larvae are visual impaired already in an early stage of life. Subsequently, optokinetic responses were measured using an in-house experimental set-up. The number of saccades was used as a measure for the optokinetic response. No significant difference in the number of eye movements between wild-type and *eys*^*rmc101/rmc101*^ larvae was observed (*p* = 0.9710, n = 19) (Fig 3E).

### Impaired locomotor activity of *eys*^*rmc101/rmc101*^ zebrafish in response to light

**Fig 4:**
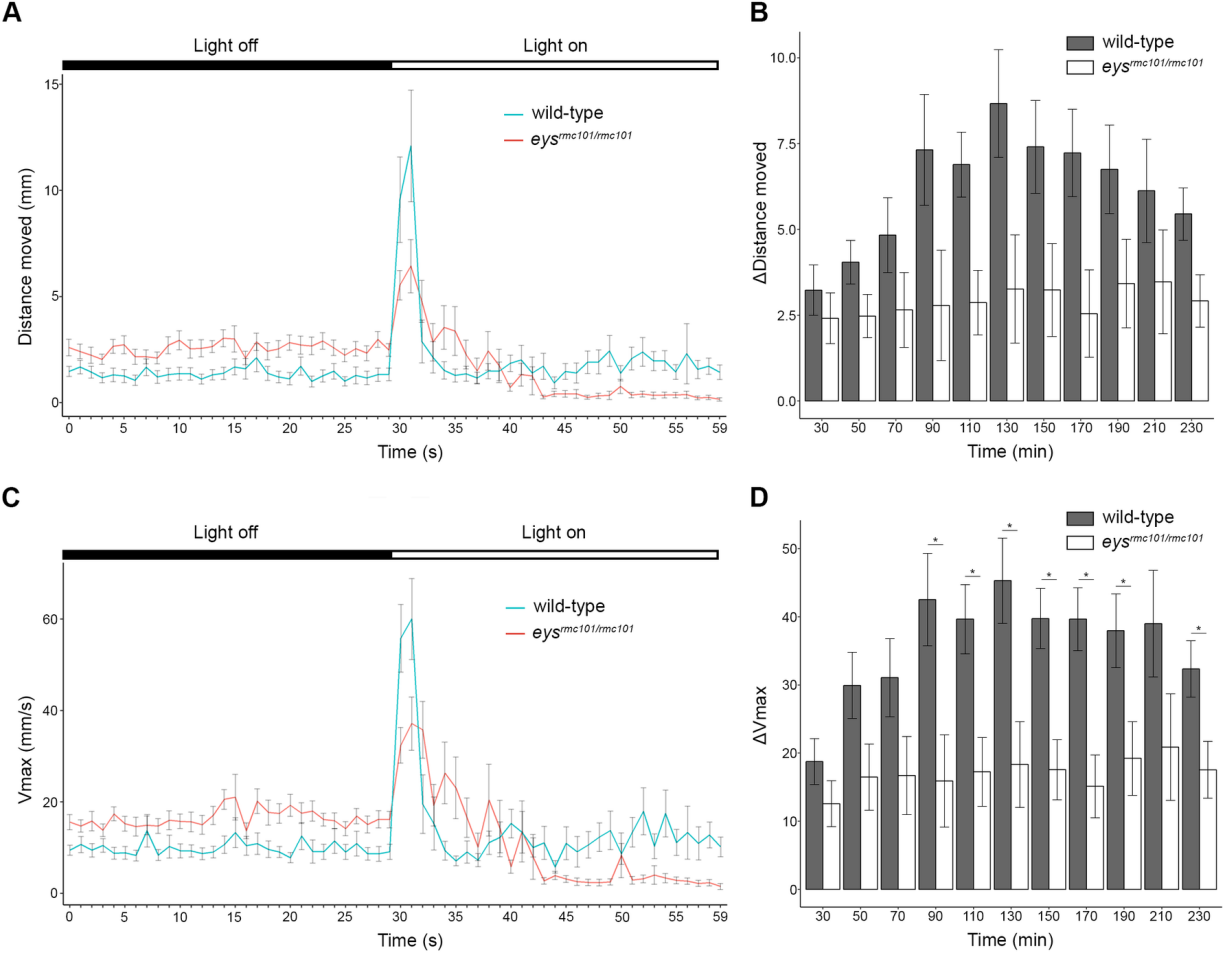
Visual motor response of zebrafish larvae. **(A)** Distance moved (mm) of wild-type (blue line) and *eys*^*rmc101/rmc101*^ (red line) larvae in response to a light stimulus (dark-to-light transition at t = 50 minutes). **(B)** Comparison of difference in distance moved between wild-type and *eys*^*rmc101/rmc101*^ larvae at the dark to light transition zones. **(C)** Maximum velocity (Vmax; mm/s) of wild-type (blue line) and *eys*^*rmc101/rmc101*^ (red line) larvae in response to a light stimulus (dark-to-light transition at t = 50 minutes). **(D)** Comparison of difference in Vmax between wild-type and *eys*^*rmc101/rmc101*^ larvae at the dark to light transition zones. All experiments were done with larvae at 5 dpf (n = 120). Statistical significance (*p*<0.05) is indicated with an asterisk.

Locomotor activity of wild-type and *eys*^*rmc101/rmc101*^ larvae (n = 120) in response to a light stimulus, the visual motor response (VMR), was examined using the Danio Vision system. Larvae were exposed to alternating periods of 10 minutes dark and 10 minutes bright light for 12 cycles in total and parameters of interest were the distance moved and the maximum velocity (Vmax). We specifically analyzed these parameters during the transition from dark-to-light (last 30 seconds of dark period and first 30 seconds of light period), since this is described to contain the visual startle response [16, 17]. Both wild-type and mutant larvae start to move at the moment the light is switched on. However, the response to light was less pronounced in the *eys* knock-out larvae compared to the wild-type larvae (Fig 4A and 4C). Furthermore, we analyzed the change in distance moved and the change in Vmax during the transition from dark-to-light, since the baseline activity of wild-type and *eys*^*rmc101/rmc101*^ fish were not equal. Overall, our data showed that the difference in Vmax was decreased in *eys*^*rmc101/rmc101*^ larvae compared to wild-type larvae and that this decreased response was significantly lower at seven out of twelve dark-to-light transition zones (Fig 4D). Similarly, we observed a major decrease of the difference in distance moved in *eys*^*rmc101/rmc101*^ fish compared to wild-type fish for all dark-to-light transition zones (Fig 4B), though not significantly different. These data show that there is a diminished response to light by *eys* mutant larvae compared to age- and strain-matched wild-type larvae.

## Discussion

Mutations in *EYS* are among the most frequent causes of arRP, yet the exact retinal function of EYS and the pathogenic mechanism underlying *EYS*-associated RP are still poorly understood. In this study, we characterized *eys* in zebrafish and showed that absence of Eys leads to disorganization of the photoreceptor cell layer and functional impairment of the zebrafish retina. Furthermore, we showed decreased locomotor activity in response to light stimuli in *eys*^*rmc101/rmc101*^ knock-out zebrafish larvae compared to wild-type larvae.

The zebrafish Eys protein lacks the low-complexity region as observed in human EYS protein, in which no protein domains are predicted. In human, this region is completely encoded by exon 26 of *EYS*. When analyzing the cDNA sequence of zebrafish *eys*, no corresponding exon of human *EYS* exon 26 could be found. In our multiple sequence alignment, one can also observe that this low complexity region is also missing from chicken EYS. This might suggest that the low complexity region, as observed in human EYS, is not of great importance for EYS to be a functional protein, at least not in all vertebrate species. In addition, a blast search with the amino acid sequence of this region only gave hits of human EYS protein and a number of EYS orthologs, such as gorilla, macaque, pig and dog.

A role of *Eys* in the morphogenesis of photoreceptors is described in *Drosophila* [7]. In the human retina, EYS might play a role in photoreceptor survival, however, certain rodent species including mouse, rat and guinea pig completely lack the *Eys* gene [6]. This raises the interesting questions on how photoreceptors in these species can survive.

In humans, retinitis pigmentosa is characterized by the progressive degeneration of rod photoreceptor cells, and in a later stage also cone photoreceptor cells. In our study, we also observed degeneration of photoreceptor outer segments in our zebrafish *eys*^*rmc101/rmc101*^ knock-out model. It is remarkable that the *eys*^*rmc101/rmc101*^ zebrafish show this clear morphological phenotype, since it is known that retinal regeneration can occur in zebrafish [18]. The Müller glia cells in zebrafish have the capacity to reprogram into retinal stem cells in response to injury, in contrast to mammals, where Müller glia cells rarely divide [19]. We investigated the presence of Müller glia activation in *eys*^*rmc101/rmc101*^ zebrafish by immunohistochemistry using an antibody against GFAP. This specific cytoskeletal protein is shown to be upregulated in response to different types of retinal injury, such as mechanical damage and photoreceptor degeneration [15, 20, 21]. In our study, we did not observe any Müller glia activation by immunohistochemistry on retinal sections of wild-type and *eys* mutant fish (5 dpf and 2 mpf). It is possible that Müller glia activation occurs in later stages when degeneration is more severe. Another explanation could be that at the protein level, Müller glia activaton in this stage of degeneration is not detectable yet. Studying gene expression levels in Müller glia cells might be a good alternative, since it is shown that these levels change rapidly after photoreceptor loss [22]. However, our data suggests that the regeneration capacity in the zebrafish is not sufficient to overcome outer segment degeneration caused by absence of Eys.

Rhodopsin, which is located in the outer segments of rod photoreceptors, is a light-sensitive receptor protein and plays a major role in visual phototransduction cascade. In the absence of Eys, rhodopsin was mislocalized to the photoreceptor inner segments and synapses. An explanation for the mislocalization of rhodopsin might be that the absence of Eys directly affects the transport of rhodopsin to the photoreceptor outer segments. Another reason could be that rhodopsin is not able to reach the outer segments of the photoreceptor as a consequence of the morphological changes within *eys*^*rmc101/rmc101*^ zebrafish. A previous study by Lu et al. also showed mislocalization of rhodopsin in *eys*^*-/-*^ zebrafish retinas, as well as mislocalization of two other outer segment proteins, guanine nucleotide-binding protein G(I)/G(S)/G(T) subunit beta-3 (GNB3) and peripherin-2 (PRPH2) [9]. Besides the peripheral mislocalization of rhodopsin, we also observed mislocalization of cone transducin (GNAT2). Next to that, GNAT2 immunofluorescence was decreased in *eys*^*rmc101/rmc101*^ retinas compared to wild-type retinas, indicating mislocalization of GNAT2 or degeneration of cone photoreceptors which is in agreement with previous studies of both Yu et al. and Lu et al. that show degeneration of cone photoreceptor cells in their *eys*^*-/-*^ zebrafish lines [9, 10].

In line with the paper of Yu et al. [10], we showed that Eys localizes in the region of the photoreceptor connecting cilium. Two immunofluorescent dots of Eys were observed together near the centrin signal, suggesting that Eys might localize in the ciliary basal body and the daughter centriole. These data also implicate a possible role of Eys in facilitating ciliary transport of proteins towards the base of the cilium. To be able to further investigate the function of EYS it will be important to know the exact localization of eys in the ciliary zone, for instance by performing electromicroscopy studies.

To determine the effect of *eys* knock-out on the visual function of the zebrafish larvae (5dpf), we performed OKR, ERG and VMR experiments. No differences in OKR were observed between wild-type and mutant zebrafish larvae; however, ERG and VMR experiments showed that *eys*^*rmc101/rmc101*^ zebrafish were visually impaired. A reason why we did not see any difference in OKR between wild-type and mutant zebrafish might be that this assay was not sensitive enough. In *eys*^*rmc101/rmc101*^ larvae, the VMR was significantly decreased compared to wild-type larvae. When analyzing the data of the VMR experiments, we specifically focused on the transition between light and dark, which is shown to be the eye-specific startle response [16, 17]. The light-off response is not only driven by photoreceptors in the retina, but also deep brain photoreceptors play an important role in this process [23, 24]. Electroretinography data showed a significantly diminished b-wave amplitude in *eys* mutant fish compared to age- and strain-matched wild-type fish, however, no difference was observed for the a-wave amplitude between mutant and wild-type fish. The a-wave is a result of photoreceptor activity, whereas the b-wave mainly reflects depolarization of ON bipolar cells [25]. The dimished b-wave amplitude in *eys*^*rmc101/rmc101*^ zebrafish could either be the result of a defect of the ON bipolar cells itself, or the signal transduction towards the ON bipolar cells. Since in zebrafish of 5 dpf, rod photoreceptors are not fully functional yet [26, 27], the normal a-wave response indicates that the cone photoreceptors of larvae at least partially remain their function in the absence of Eys.

Since *EYS* is not present in several rodent species, we used zebrafish to study the function of Eys *in vivo*. Furthermore, the morphology of the zebrafish retina is similar to that of the human retina, in the sense that all the major cell layers found in humans are also present in zebrafish. Another approach to further study the function of EYS and the pathogenic mechanism underlying *EYS*-related RP, would be to make use of patient-derived cells. Due to the retina-specific expression of *EYS*, one would have to use induced pluripotent stem cells (iPSCs), which can be differentiated into retinal cells [28, 29]. Cells derived from patients with *EYS* mutations can be compared to cells derived from controls, by looking at gene expression levels, EYS localization, and morphological differences.

In conclusion, we generated an *eys*^*rmc101/rmc101*^ zebrafish line, which showed disorganization of the photoreceptor outer segments and impaired visual function. Furthermore, absence of Eys leads to a significantly decreased VMR, which was not shown previously. In addition, this *eys*^*rmc101/rmc101*^ zebrafish line is a powerful model to further study the pathophysiological mechanism underlying *EYS*-associated RP.

## Supporting information

S1 Table Primers used for PCR analysis. (DOCX)

S2 Table Oligo sequences used for gRNA synthesis. (DOCX)

S3 Table P-values of VMR experiment. (DOCX)

S1 Fig Gene predictions, exons and protein domain structure. **(A)** Upper panel: gene predictions present for zebrafish *eys* in the UCSC Genome Browser on chromosome 13. Lower panel: Exon structure of zebrafish *eys*. **(B)** Protein domain structure of human EYS and zebrafish Eys proteins. Note the conservation of Laminin G domains at the C-terminal part of the protein.
(TIF)

S2 Fig Multiple sequence alignment of EYS protein in different species. Residues identical in all sequences are white on a black background, whereas similar amino acids are white on a gray background. Residues that are present in at least three of the five proteins are indicated in black on a light gray background. Accession numbers of the protein sequences used for sequence comparison are as follows: human, NM_001142800.1 (RefSeq); macaque, XM_011737495.1 (RefSeq); chicken, XM_015284845.1 (RefSeq); *Drosophila*, NM_001032399.3 (RefSeq).
(PDF)

S3 Fig Measurement of the thickness of outer and inner nuclear layers in *eys*^*rmc101/rmc101*^ and wild-type zebrafish. **(A, C, E)** Representative images of wild-type and *eys*^*rmc101/rmc101*^ zebrafish retinas at (A) 5 dpf, (C) 2 mpf, and (E) 5 mpf. Nuclear layers were stained with DAPI and inverted to grey images. ONL: outer nuclear layer; OPL: outer plexiform layer; INL: inner nuclear layer; IPL: inner plexiform layer; GCL: ganglion cell layer. Scale bars (A): 10 μm. Scale bars (C, E): 20 μm. **(B, D, F)** Measurements of ONL and INL thickness in wild-type and *eys*^*rmc101/rmc101*^ zebrafish at (A) 5 dpf, (C) 2 mpf, and (E) 5 mpf. Asterisks indicate statistical significance (*** = *p*<0.0001) using Mann-Whitney U test.
(TIF)

S4 Fig Immunohistochemistry on retinal sections of wild-type and *eys*^*rmc101/rmc101*^ zebrafish in order to investigate Müller glia activation. Retinal sections of wild-type and *eys*^*rmc101/rmc101*^ zebrafish at 5 dpf and 2 mpf stained with antibodies against GFAP (green), as a marker for Müller glia cells. Müller glia cell bodies are located in the inner nuclear layer (arrow heads) and project processes (arrows) in either direction to outer limiting membrane and inner limiting membrane. Nuclei are counterstained with DAPI (blue). INL: inner nuclear layer; IPL: inner plexiform layer; ONL: outer nuclear layer. Scale bar: 20 μm.
(TIF)

## References

[1] Hartong DT, Berson EL, Dryja TP. Retinitis pigmentosa. Lancet. 2006;368(9549):1795–809. 10.1016/S0140-6736(06)69740-7 . 17113430

[2] Littink KW, van den Born LI, Koenekoop RK, Collin RW, Zonneveld MN, Blokland EA, et al Mutations in the EYS gene account for approximately 5% of autosomal recessive retinitis pigmentosa and cause a fairly homogeneous phenotype. Ophthalmology. 2010;117(10):2026–33, 33 e1–7. 10.1016/j.ophtha.2010.01.040 . 20537394

[3] Barragan I, Borrego S, Pieras JI, Gonzalez-del Pozo M, Santoyo J, Ayuso C, et al Mutation spectrum of EYS in Spanish patients with autosomal recessive retinitis pigmentosa. Hum Mutat. 2010;31(11):E1772–800. 10.1002/humu.21334 . 21069908 PMC3045506

[4] Hosono K, Ishigami C, Takahashi M, Park DH, Hirami Y, Nakanishi H, et al Two novel mutations in the EYS gene are possible major causes of autosomal recessive retinitis pigmentosa in the Japanese population. PloS one. 2012;7(2):e31036 10.1371/journal.pone.0031036 . 22363543 PMC3281914

[5] Collin RW, Littink KW, Klevering BJ, van den Born LI, Koenekoop RK, Zonneveld MN, et al Identification of a 2 Mb human ortholog of Drosophila eyes shut/spacemaker that is mutated in patients with retinitis pigmentosa. American journal of human genetics. 2008;83(5):594–603. 10.1016/j.ajhg.2008.10.014 . 18976725 PMC2668042

[6] Abd El-Aziz MM, Barragan I, O’Driscoll CA, Goodstadt L, Prigmore E, Borrego S, et al EYS, encoding an ortholog of Drosophila spacemaker, is mutated in autosomal recessive retinitis pigmentosa. Nature genetics. 2008;40(11):1285–7. 10.1038/ng.241 . 18836446 PMC2719291

[7] Husain N, Pellikka M, Hong H, Klimentova T, Choe KM, Clandinin TR, et al The agrin/perlecan-related protein eyes shut is essential for epithelial lumen formation in the Drosophila retina. Developmental cell. 2006;11(4):483–93. 10.1016/j.devcel.2006.08.012 . 17011488

[8] Slijkerman RW, Song F, Astuti GD, Huynen MA, van Wijk E, Stieger K, et al The pros and cons of vertebrate animal models for functional and therapeutic research on inherited retinal dystrophies. Progress in retinal and eye research. 2015;48:137–59. 10.1016/j.preteyeres.2015.04.004 . 25936606

[9] Lu Z, Hu X, Liu F, Soares DC, Liu X, Yu S, et al Ablation of EYS in zebrafish causes mislocalisation of outer segment proteins, F-actin disruption and cone-rod dystrophy. Sci Rep. 2017;7:46098 10.1038/srep46098 . 28378834 PMC5380955

[10] Yu M, Liu Y, Li J, Natale BN, Cao S, Wang D, et al Eyes shut homolog is required for maintaining the ciliary pocket and survival of photoreceptors in zebrafish. Biol Open. 2016;5(11):1662–73. 10.1242/bio.021584 . 27737822 PMC5155541

[11] Montague TG, Cruz JM, Gagnon JA, Church GM, Valen E. CHOPCHOP: a CRISPR/Cas9 and TALEN web tool for genome editing. Nucleic acids research. 2014;42(Web Server issue):W401–7. 10.1093/nar/gku410 . 24861617 PMC4086086

[12] Gagnon JA, Valen E, Thyme SB, Huang P, Akhmetova L, Pauli A, et al Efficient mutagenesis by Cas9 protein-mediated oligonucleotide insertion and large-scale assessment of single-guide RNAs. PloS one. 2014;9(5):e98186 10.1371/journal.pone.0098186 . 24873830 PMC4038517

[13] Huber-Reggi SP, Mueller KP, Neuhauss SC. Analysis of optokinetic response in zebrafish by computer-based eye tracking. Methods Mol Biol. 2013;935:139–60. 10.1007/978-1-62703-080-9_10 . 23150366

[14] Sirisi S, Folgueira M, Lopez-Hernandez T, Minieri L, Perez-Rius C, Gaitan-Penas H, et al Megalencephalic leukoencephalopathy with subcortical cysts protein 1 regulates glial surface localization of GLIALCAM from fish to humans. Hum Mol Genet. 2014;23(19):5069–86. 10.1093/hmg/ddu231 . 24824219

[15] Chen H, Weber AJ. Expression of glial fibrillary acidic protein and glutamine synthetase by Muller cells after optic nerve damage and intravitreal application of brain-derived neurotrophic factor. Glia. 2002;38(2):115–25 . 11948805 10.1002/glia.10061

[16] Burgess HA, Granato M. Modulation of locomotor activity in larval zebrafish during light adaptation. J Exp Biol. 2007;210(Pt 14):2526–39. 10.1242/jeb.003939 . 17601957

[17] Easter SS Jr., Nicola GN. The development of vision in the zebrafish (Danio rerio). Dev Biol. 1996;180(2):646–63 . 8954734 10.1006/dbio.1996.0335

[18] Wan J, Goldman D. Retina regeneration in zebrafish. Curr Opin Genet Dev. 2016;40:41–7. 10.1016/j.gde.2016.05.009 . 27281280 PMC5135611

[19] Goldman D. Muller glial cell reprogramming and retina regeneration. Nat Rev Neurosci. 2014;15(7):431–42. 10.1038/nrn3723 . 24894585 PMC4249724

[20] Nagashima M, Barthel LK, Raymond PA. A self-renewing division of zebrafish Muller glial cells generates neuronal progenitors that require N-cadherin to regenerate retinal neurons. Development. 2013;140(22):4510–21. 10.1242/dev.090738 . 24154521 PMC3817940

[21] de Raad S, Szczesny PJ, Munz K, Reme CE. Light damage in the rat retina: glial fibrillary acidic protein accumulates in Muller cells in correlation with photoreceptor damage. Ophthalmic Res. 1996;28(2):99–107. 10.1159/000267881 . 8792360

[22] Sifuentes CJ, Kim JW, Swaroop A, Raymond PA. Rapid, Dynamic Activation of Muller Glial Stem Cell Responses in Zebrafish. Invest Ophthalmol Vis Sci. 2016;57(13):5148–60. 10.1167/iovs.16-19973 . 27699411 PMC5054728

[23] Fernandes AM, Fero K, Arrenberg AB, Bergeron SA, Driever W, Burgess HA. Deep brain photoreceptors control light-seeking behavior in zebrafish larvae. Curr Biol. 2012;22(21):2042–7. 10.1016/j.cub.2012.08.016 . 23000151 PMC3494761

[24] Ganzen L, Venkatraman P, Pang CP, Leung YF, Zhang M. Utilizing Zebrafish Visual Behaviors in Drug Screening for Retinal Degeneration. Int J Mol Sci. 2017;18(6). 10.3390/ijms18061185 . 28574477 PMC5486008

[25] Fleisch VC, Jametti T, Neuhauss SC. Electroretinogram (ERG) Measurements in Larval Zebrafish. CSH Protoc. 2008;2008:pdb prot4973. 10.1101/pdb.prot4973 . 21356789

[26] Morris AC, Fadool JM. Studying rod photoreceptor development in zebrafish. Physiol Behav. 2005;86(3):306–13. 10.1016/j.physbeh.2005.08.020 . 16199068 PMC2810101

[27] Branchek T, Bremiller R. The development of photoreceptors in the zebrafish, Brachydanio rerio. I. Structure. J Comp Neurol. 1984;224(1):107–15. 10.1002/cne.902240109 . 6715574

[28] Zhong X, Gutierrez C, Xue T, Hampton C, Vergara MN, Cao LH, et al Generation of three-dimensional retinal tissue with functional photoreceptors from human iPSCs. Nat Commun. 2014;5:4047 10.1038/ncomms5047 . 24915161 PMC4370190

[29] Tucker BA, Mullins RF, Streb LM, Anfinson K, Eyestone ME, Kaalberg E, et al Patient-specific iPSC-derived photoreceptor precursor cells as a means to investigate retinitis pigmentosa. Elife. 2013;2:e00824 10.7554/eLife.00824 . 23991284 PMC3755341

