# Supplementary material for: Eyes shut homolog is important for the maintenance of photoreceptor morphology and visual function in zebrafish
Source: PLoS One. 2018 Jul 27;13(7):e0200789. doi: 10.1371/journal.pone.0200789 (PMC6063403; doi:10.1371/journal.pone.0200789)
Supplement: S1 Table — (DOCX) [file pone.0200789.s001.docx]

**S1 Table.** **Primers used for PCR analysis.**

| **Purpose** | **Product** | **Sequence (5’ > 3’)** | **Comment** |
| --- | --- | --- | --- |
| Sequence analysis | *eys* exon 1-6 forward | TGAGGGAAGTGCCTACACAG |  |
|  | *eys* exon 1-6 forward | GTCTTTGTCCTGCTCCATTC | Nested |
|  | *eys* exon 1-6 reverse | GCAAGTAGCTCCGTTCAAGC |  |
|  | *eys* exon 1-6 reverse | GATAGGCTAAGCATGCGTTC | Nested |
|  | *eys* exon 6-10 forward | CTGCACCCCCGCATTATATG |  |
|  | *eys* exon 6-10 forward | GCACCTGTCTCCCTGGATAC | Nested |
|  | *eys* exon 6-10 reverse | CCTCATATCCCAGCATGCAG |  |
|  | *eys* exon 6-10 reverse | TAGTTGTGCTGTTGGTCCAC | Nested |
|  | *eys* exon 10-17 forward | TGATTTGGTGGCAGGATAC |  |
|  | *eys* exon 10-17 forward | ATACCAATGCCTGTGTGCAC | Nested |
|  | *eys* exon 10-17 reverse | ACACACTCTGCCCCGTTATC |  |
|  | *eys* exon 10-17 reverse | GTTATCGCACGGATTAACAGC | Nested |
|  | *eys* exon 18-20 forward | TAACCCTGGTTACGCTGGAG |  |
|  | *eys* exon 18-20 forward | TCAATGACTGCGCTAGCAAC | Nested |
|  | *eys* exon 18-20 reverse | AAACTCCCCTGGGGTATCAG |  |
|  | *eys* exon 18-20 reverse | TCCATCCTTGAGGACACACA | Nested |
|  | *eys* exon 20-22 forward | GATACCCCAGGGGAGTTTTC |  |
|  | *eys* exon 20-22 forward | ACAGCCCTATGACCCTTGTG | Nested |
|  | *eys* exon 20-22 reverse | GATGCGCATTCATTGATGTC |  |
|  | *eys* exon 20-22 reverse | ACAGTGCAGGCCTGAAAATC | Nested |
|  | *eys* exon 21-25 forward | GTGAAATTGATAGCGATGACTG |  |
|  | *eys* exon 21-25 forward | TGGATGGGGTCAACAGCTAC | Nested |
|  | *eys* exon 21-25 reverse | CAACTCCCATGAACACAGGG |  |
|  | *eys* exon 21-25 reverse | TGTTTCTCTCGCAGTGATCG | Nested |
|  | *eys* exon 24-28 forward | TCGACGGCCTTAATGGTTAC |  |
|  | *eys* exon 24-28 forward | TCACTGCGAGAGAAACACTG | Nested |
|  | *eys* exon 24-28 reverse | CAGGCTCATTCTGGATGCAC |  |
|  | *eys* exon 24-28 reverse | ATTCCCTGGTTGCATGGTTG | Nested |
|  | *eys* exon 28-32 forward | GGGAATGTGCATCCAGAATG |  |
|  | *eys* exon 28-32 forward | TGGCTACACCTGCTTCTGTC | Nested |
|  | *eys* exon 28-32 reverse | TCCTGACACCACTAACGGTG |  |
|  | *eys* exon 28-32 reverse | TCAGCCACACACGGAAAAAG | Nested |
|  | *eys* exon 32-36 forward | CCATGTGTTTATTGGAGGTC |  |
|  | *eys* exon 32-36 forward | CTCGTCATCGTTCACCTTAC | Nested |
|  | *eys* exon 32-36 reverse | CTTGCATTCTGGAGAAACAC |  |
|  | *eys* exon 32-36 reverse | AGAAAACGCTCTCCCAAATC | Nested |
|  | *eys* exon 36-39 forward | TGGCTGCTCAGAATATTAGGATC |  |
|  | *eys* exon 36-39 forward | GATAGTTTGGTGGCAATTACAGTG | Nested |
|  | *eys* exon 36-39 reverse | GGCACAGCTTACCGGAGTAG |  |
|  | *eys* exon 36-39 reverse | CATGCACAGAACCAGCTCTC | Nested |
|  | *eys* exon 39-40 forward | TGGTTCTGTGCATGTCCTTC |  |
|  | *eys* exon 39-40 forward | CGCTCTACTCCGGTAAGCTG | Nested |
|  | *eys* exon 39-40 reverse | AGGCATTATTGGCAAAGGTG |  |
|  | *eys* exon 39-40 reverse | GGGCCGAGTGATGTTGATAG | Nested |
|  | *eys* exon 40-44 forward | TCACCTGAAGCTCACCTTTG |  |
|  | *eys* exon 40-44 forward | TTCTCTTTTCCGGGCAAAAG | Nested |
|  | *eys* exon 40-44 reverse | CAGAACGACACTTTCTCTGAGC |  |
|  | *eys* exon 40-44 reverse | GCACCTTTCCATCCAAACAC | Nested |
|  | *eys* exon 44-46 forward | GTGTTTGGATGGAAAGGTGC |  |
|  | *eys* exon 44-46 forward | GCTCAGAGAAAGTGTCGTTCTG | Nested |
|  | *eys* exon 44-46 reverse | TTGAATGGACTGTTGGCATC |  |
|  | *eys* exon 44-46 reverse | CCCAGATTATAGCGGAGCTG | Nested |
|  | *eys* exon 46 forward | TAAGGTTTGCAAGAACGGTG |  |
|  | *eys* exon 46 forward | TGATCATCTAGCGGTTGGAC | Nested |
|  | *eys* exon 46 reverse | ACTCTTCTCCTTGATAGACATTGAAAC |  |
|  | *eys* exon 46 reverse | TGGATGAGAGTCCGATTGTG | Nested |
| RT-PCR analysis | *eys* exon 19-21 forward | CTGCACAGACCTCCTCAATG |  |
|  | *eys* exon 19-21 reverse | CACAGCGAGTTCCCTCAAAC |  |
|  | *actin* forward | CAACAGGGAAAAGATGACACAGAT |  |
|  | *actin* reverse | CAGCCTGGATGGCAACGT |  |
| Genomic lesions | *eys* exon 20 forward | TGGGTAAAGAATGCCCAACT | In intron 19 |
|  | *eys* exon 20 reverse | GCAAACAAAGCCACAGAACA | In intron 20 |
