# Supplementary material for: Eyes shut homolog is important for the maintenance of photoreceptor morphology and visual function in zebrafish
Source: PLoS One. 2018 Jul 27;13(7):e0200789. doi: 10.1371/journal.pone.0200789 (PMC6063403; doi:10.1371/journal.pone.0200789)
Supplement: S2 Table — (DOCX) [file pone.0200789.s002.docx]

**S2 Table.** **Oligo sequences used for gRNA synthesis.**

| **Oligo name** | **Sequence** |
| --- | --- |
| Constant oligo | AAAAGCACCGACTCGGTGCCACTTTTTCAAGTTGATAACGGACTAGCCTTATTTTAACTTGCTATTTCTAGCTCTAAAAC |
| Gene specific oligo *eys* exon 20 | **TAATACGACTCACTATAGGTGCAGGAAAACTCCCCTG**GTTTTAGAGCTAGAAATAGCAAG |

T7 promoter in bold. Gene specific region is red and underlined. Overlapping region is underlined.
