# Supplementary material for: Eyes shut homolog is important for the maintenance of photoreceptor morphology and visual function in zebrafish
Source: PLoS One. 2018 Jul 27;13(7):e0200789. doi: 10.1371/journal.pone.0200789 (PMC6063403; doi:10.1371/journal.pone.0200789)
Supplement: S3 Table — (DOCX) [file pone.0200789.s003.docx]

**S3 Table. P-values of VMR experiment.**

P-values of **(A)** Δ distance moved (mm) and **(B)** ΔVmax (mm/s). R programming language was used to generate plots, calculate mean values and SEM values, and perform statistical tests. The difference between wild-type and mutant was analyzed using two-tailed, unpaired Student’s *t*-test, and *p*-values were corrected for multiple testing using the Benjamini-Hochberg method.

**A**

| **Time (min)** | **Exp. 1 – average wild-type** | **Exp. 2 – average wild-type** | **Exp. 3 – average wild-type** | **Exp. 4 – average wild-type** | **Exp. 5 – average wild-type** | **Exp. 1 – average mutant** | **Exp. 2 – average mutant** | **Exp. 3 – average mutant** | **Exp. 4 – average mutant** | **Exp. 5 – average mutant** | **p-value** | **Adjust. p-value** |
| --- | --- | --- | --- | --- | --- | --- | --- | --- | --- | --- | --- | --- |
| **30** | 4.8197 | 2.0511 | 3.4826 | 1.0884 | 4.7208 | 2.5904 | 3.4860 | 1.4284 | 2.4223 | 2.1277 | 0.3507 | 0.3507 |
| **50** | 4.2654 | 1.6485 | 5.3231 | 4.2653 | 4.7361 | 2.5555 | 2.8459 | 1.8697 | 1.7964 | 3.3079 | 0.0669 | 0.0920 |
| **70** | 6.1754 | 3.1097 | 2.1892 | 4.4411 | 8.2672 | 2.6798 | 4.7682 | 1.6366 | 1.8173 | 2.3796 | 0.1255 | 0.1533 |
| **90** | 8.2053 | 2.9419 | 7.0583 | 5.6787 | 12.7090 | 2.9766 | 4.6111 | 2.1596 | 1.2555 | 2.9299 | 0.0453 | 0.0830 |
| **110** | 7.7536 | 4.9493 | 7.9612 | 4.4440 | 9.3504 | 2.7831 | 4.8332 | 2.2791 | 2.0060 | 2.4370 | 0.0089 | 0.0743 |
| **130** | 11.4764 | 3.6879 | 11.1431 | 6.1969 | 10.8313 | 3.2687 | 5.7597 | 2.8176 | 1.6177 | 2.8589 | 0.0226 | 0.0743 |
| **150** | 8.5364 | 4.0738 | 10.9260 | 4.3820 | 9.1149 | 3.2371 | 3.9485 | 2.7486 | 1.2618 | 4.9879 | 0.0338 | 0.0743 |
| **170** | 8.5113 | 4.7271 | 10.9045 | 4.0220 | 7.9785 | 2.9695 | 3.0634 | 2.4767 | 1.7028 | 2.5132 | 0.0198 | 0.0743 |
| **190** | 9.5821 | 2.9989 | 9.3845 | 4.7361 | 7.0626 | 3.4548 | 2.5366 | 2.6767 | 2.7026 | 5.7391 | 0.0601 | 0.0920 |
| **210** | 5.5026 | 2.5350 | 11.7022 | 5.5734 | 5.3175 | 4.8168 | 3.8193 | 2.6527 | 2.3314 | 3.7506 | 0.1558 | 0.1714 |
| **230** | 6.6847 | 3.1552 | 7.1342 | 6.0788 | 4.2068 | 3.0005 | 2.5957 | 1.8931 | 2.3267 | 4.7831 | 0.0275 | 0.0743 |

**B**

| **Time (min)** | **Exp. 1 – average wild-type** | **Exp. 2 – average wild-type** | **Exp. 3 – average wild-type** | **Exp. 4 – average wild-type** | **Exp. 5 – average wild-type** | **Exp. 1 – average mutant** | **Exp. 2 – average mutant** | **Exp. 3 – average mutant** | **Exp. 4 – average mutant** | **Exp. 5 – average mutant** | **p-value** | **Adjust. p-value** |
| --- | --- | --- | --- | --- | --- | --- | --- | --- | --- | --- | --- | --- |
| **30** | 24.576 | 12.363 | 22.723 | 9.038 | 25.130 | 12.611 | 15.315 | 10.776 | 13.036 | 11.128 | 0.1400 | 0.1400 |
| **50** | 26.202 | 14.173 | 43.759 | 33.043 | 32.502 | 13.241 | 24.963 | 10.354 | 9.197 | 24.620 | 0.0571 | 0.0784 |
| **70** | 39.481 | 20.473 | 18.155 | 28.830 | 48.366 | 16.741 | 30.126 | 14.232 | 9.437 | 13.031 | 0.0724 | 0.0883 |
| **90** | 45.996 | 24.524 | 43.843 | 33.485 | 64.756 | 16.001 | 27.289 | 10.495 | 7.482 | 18.337 | 0.0129 | 0.0284 |
| **110** | 45.805 | 31.141 | 43.932 | 24.936 | 52.443 | 13.165 | 34.066 | 13.641 | 9.813 | 15.674 | 0.0101 | 0.0277 |
| **130** | 53.787 | 28.304 | 51.908 | 32.431 | 60.032 | 18.533 | 27.993 | 17.557 | 11.794 | 15.826 | 0.0093 | 0.0277 |
| **150** | 47.229 | 27.970 | 51.420 | 32.322 | 39.784 | 16.925 | 19.967 | 18.786 | 5.851 | 26.431 | 0.0045 | 0.0246 |
| **170** | 43.461 | 29.880 | 50.023 | 27.615 | 47.284 | 19.580 | 15.348 | 15.604 | 9.500 | 15.662 | 0.0040 | 0.0246 |
| **190** | 51.698 | 21.308 | 39.062 | 31.346 | 46.442 | 21.947 | 15.285 | 16.501 | 14.173 | 28.282 | 0.0216 | 0.0339 |
| **210** | 35.533 | 20.274 | 67.925 | 36.008 | 35.274 | 24.047 | 22.713 | 17.494 | 14.651 | 25.472 | 0.0802 | 0.0883 |
| **230** | 35.456 | 20.734 | 41.845 | 39.286 | 24.518 | 17.214 | 13.480 | 12.548 | 15.774 | 28.689 | 0.0215 | 0.0339 |
