# Supplementary figures and images for: Eyes shut homolog is important for the maintenance of photoreceptor morphology and visual function in zebrafish
Source: PLoS One. 2018 Jul 27;13(7):e0200789. doi: 10.1371/journal.pone.0200789 (PMC6063403; doi:10.1371/journal.pone.0200789)

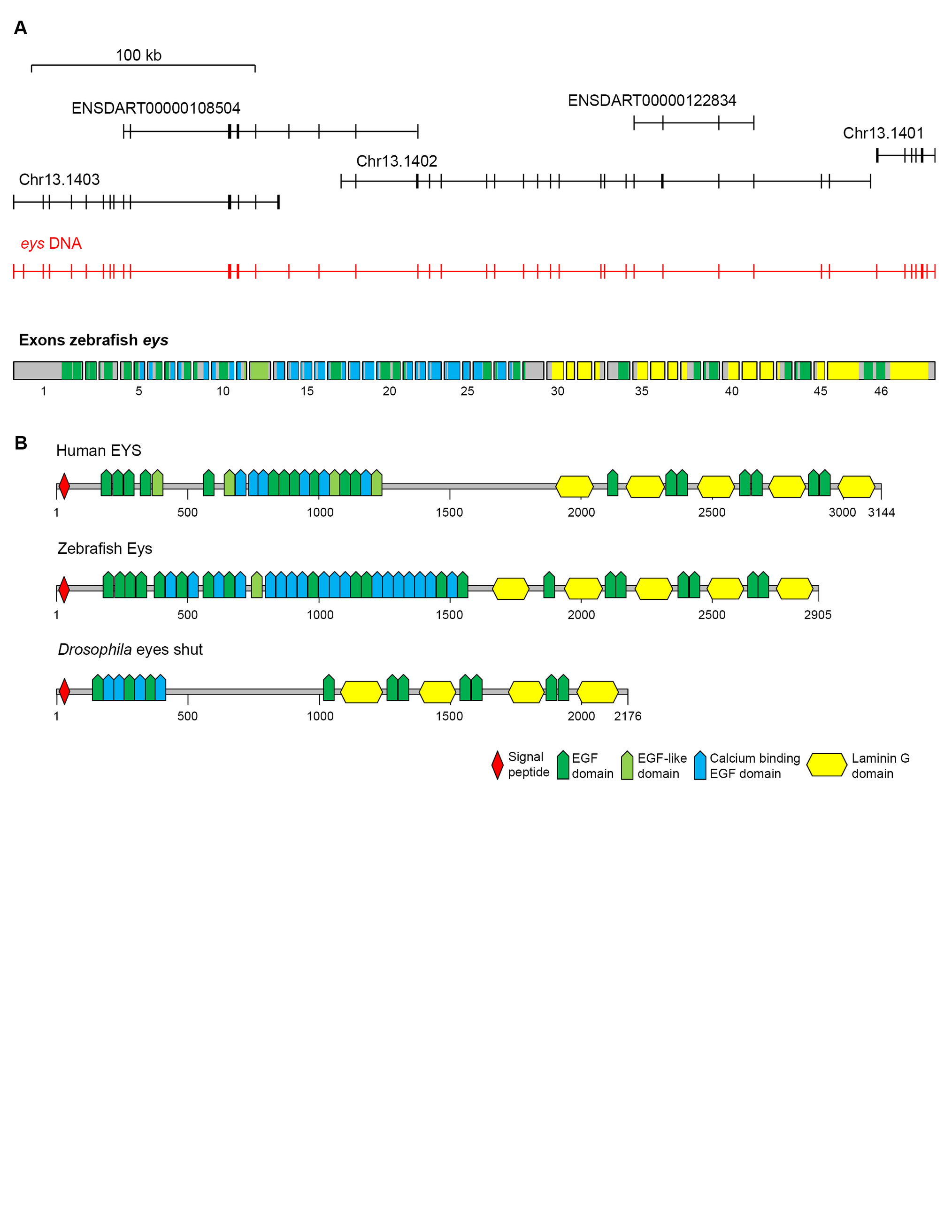

Supplement: S1 Fig — (A) Upper panel: gene predictions present for zebrafish eys in the UCSC Genome Browser on chromosome 13. Lower panel: Exon structure of zebrafish eys. (B) Protein domain structure of human EYS and zebrafish Eys proteins. Note the conservation of Laminin G domains at the C-terminal part of the protein. (TIF) [file pone.0200789.s004.tif]

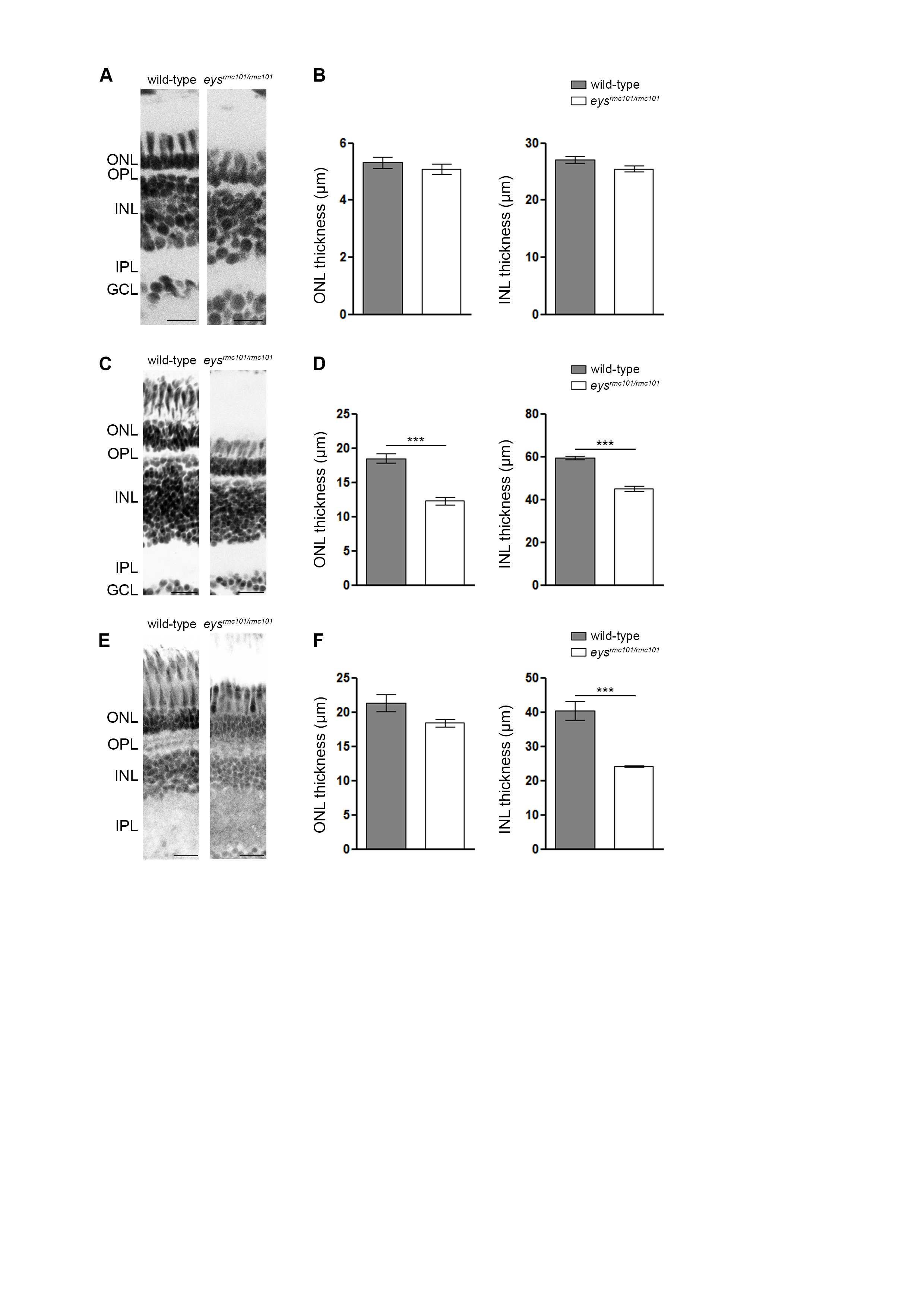

Supplement: S3 Fig — (A, C, E) Representative images of wild-type and eysrmc101/rmc101 zebrafish retinas at (A) 5 dpf, (C) 2 mpf, and (E) 5 mpf. Nuclear layers were stained with DAPI and inverted to grey images. ONL: outer nuclear layer; OPL: outer plexiform layer; INL: inner nuclear layer; IPL: inner plexiform layer; GCL: ganglion cell layer. Scale bars (A): 10 μm. Scale bars (C, E): 20 μm. (B, D, F) Measurements of ONL and INL thickness in wild-type and eysrmc101/rmc101 zebrafish at (A) 5 dpf, (C) 2 mpf, and (E) 5 mpf. Asterisks indicate statistical significance (*** = p<0.0001) using Mann-Whitney U test. (TIF) [file pone.0200789.s006.tif]

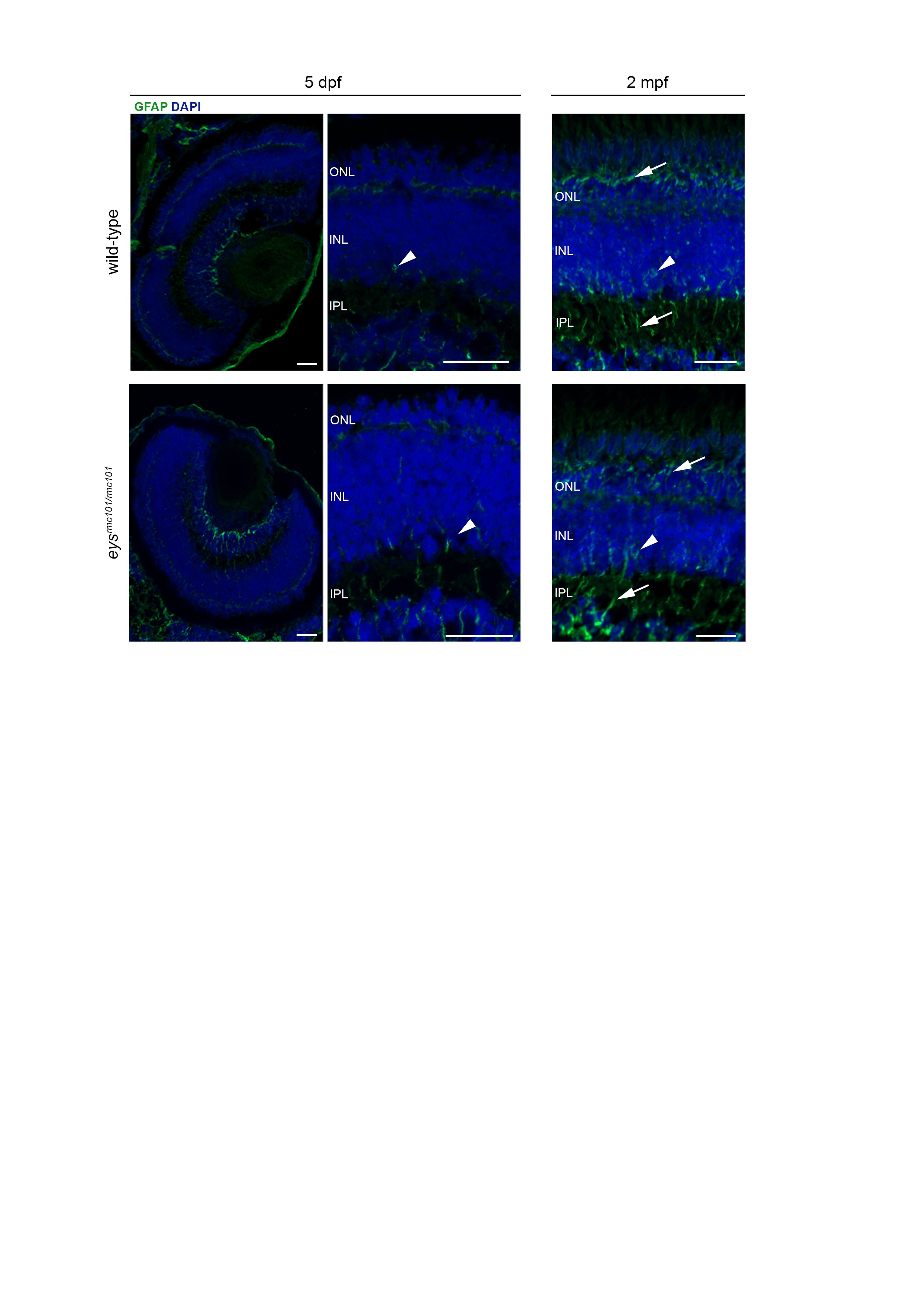

Supplement: S4 Fig — Retinal sections of wild-type and eysrmc101/rmc101 zebrafish at 5 dpf and 2 mpf stained with antibodies against GFAP (green), as a marker for Müller glia cells. Müller glia cell bodies are located in the inner nuclear layer (arrow heads) and project processes (arrows) in either direction to outer limiting membrane and inner limiting membrane. Nuclei are counterstained with DAPI (blue). INL: inner nuclear layer; IPL: inner plexiform layer; ONL: outer nuclear layer. Scale bar: 20 μm. (TIF) [file pone.0200789.s007.tif]
