# Supplementary material for: Eyes shut homolog is important for the maintenance of photoreceptor morphology and visual function in zebrafish
Source: PLoS One. 2018 Jul 27;13(7):e0200789. doi: 10.1371/journal.pone.0200789 (PMC6063403; doi:10.1371/journal.pone.0200789)
Supplement: S2 Fig — Residues identical in all sequences are white on a black background, whereas similar amino acids are white on a gray background. Residues that are present in at least three of the five proteins are indicated in black on a light gray background. Accession numbers of the protein sequences used for sequence comparison are as follows: human, NM_001142800.1 (RefSeq); macaque, XM_011737495.1 (RefSeq); chicken, XM_015284845.1 (RefSeq); Drosophila, NM_001032399.3 (RefSeq). (PDF) [file pone.0200789.s005.pdf]

S2 Fig. Multiple sequence alignment of EYS protein in different species

```
     Human      (1) --MTDKSIVILSLMVFHSSFIN-GKTCRRQLVEEWHPQPSSYVVNWTLTENICLDFYRDC
   Macaque      (1) --MTDKSIIILSLMVFHSSFIN-GKTCRRELVEEWHPQPSSHVVNWTLTENICLDYYRDC
   Chicken      (1) --MTPKSTVHMVVCFLQLCIVKGQIICERQIIAEWRVEPKHVLIEWTLRKNICTDFYSEC
 Zebrafish      (1) MRNPKLAIIVFLLSCVIYGPVYSQVTCRRATSREWHTQPKNISVRWTLMENTCSSLTQCW
Drosophila      (1) ------------------------------------------------------------
                       Signal peptide

     Human     (58) WFLGVN-TKIDTSGNQAVPQICPLQIQLGDILVISSEPSLQFPEINLMNVSETSFVGCVQ
   Macaque     (58) WFLGVN-TKIDTSGNQVVPQICPLQIQLGDILVISSEPSLQFPEINLMNVSETSFIGCVQ
   Chicken     (59) WNVNKN-ATREDKN-LSVPQICPVQLQLGDTLFISSEPSFQSYGMNLVNVSKEEFINCPK
 Zebrafish     (61) SSFAETNGHFWTTGPYHFPQLCPLELQLGDLLFVSADGTLEQHGVQLIKVSKEEFDKCAI
Drosophila      (1) ------------------------------------------------------------

     Human    (117) NTTTEDQLLFGCRLKGMHTVNSKWLSVGTHYFITVMASGPSPCPLGLRLNVTVKQQFCQE
   Macaque    (117) NTTTEDQLLFGCRLKGMHTVNSKWLSVGTHYFITVMASGPSPCPLGLRLNVTVKQQFCQE
   Chicken    (117) IGFLQEQQIFVCQIRGLHQVDSTWLGVGTHYFAELHKRGPYLCNMGLRLNVTVKQQFCQE
 Zebrafish    (121) LEPRKEQLVFASSINGTLQVESKWLMSGLNYFTIINRGSSHLCRFGLRIAVLVKPQLCQS
Drosophila      (1) ------------------------------------------------------------

     Human    (177) SLSSEFCSGHGKCLSEAWSKTYSCHCQPPFSGKYCQELDACSFKPCKNNGSCINKR-ENW
   Macaque    (177) SLSSEFCSGHGKCLSEAWSKTYSCHCQPPFSGKYCQELDACSFKPCKNDGSCINKR-GNW
   Chicken    (177) TPNAPLCSKHGRCLSRVWEKQYSCHCFSQYSGKFCQKFNVCSTKPCHNNASCTEK--LEG
 Zebrafish    (181) SPLLRLCSGKGECRTTLKDDSFTCRCHKHFSGRYCENVDGCYEQPCLNGGTCLSEGSAYT
Drosophila      (1) ------------------------------------------------------------
                           EGF domain                      EGF domain

     Human    (236) DEQAYECVCHPPFTGKNCSEIIG-QCQPHVCFHGNCSNITSNSFICECDEQFSGPFCEVS
   Macaque    (236) DEQGYECVCHPPFTGKNCSEIIV-QCQPHVCFHGNCSNITSNSFICECDEQFSGPFCEVS
   Chicken    (235) IRNSYECTCPPEFSGKNCTEIVG-QCQPHTCFNGNCSNVTPNTFLCECDKGFTGPFCEEP
 Zebrafish    (241) DLPPYTCLCPAPFTGVNCSEIIGNQNCSKWCKEGACLKVSSTSYRCECFTGYTGTYCERK
Drosophila      (1) ------------------------------------------------------------
                                                    EGF domain

     Human    (295) AKPCVSLLFWKRGICPNSSSAYTYECPKGSSSQNGETDVSEFSLVPCQNGTDCIKISNDV
   Macaque    (295) TKPCVSLLCWKRGICPNSSSAYTYECPKGPSSQNGETDVSECSLILCENGTDGIKISNDV
   Chicken    (294) GDPCASQPCLNRGVCQYNQSGYICDCPAGFLGQDCEVDINECSSRPCQNRGTCIDLPNDV
 Zebrafish    (301) RLFCDSNPCRNDGRCEETANGYVCTCPGGFTGLNCETTAEADSYCKS-SGCQLDEACATD
Drosophila      (1) ------------------------------------------------------------
                                                    EGF domain

     Human    (355) MCICSPIFTDLLCKSIQTSCESFPLRNNATCKKCEKDYPCSCISGFTEKNCEKAIDHCKL
   Macaque    (355) MCICSPIFTDLLCKSFQTSCESFPLKNNATFKKCEKDYHCSCMSGFTGKNCEKVIDHCKL
   Chicken    (354) SCSCLPLFTGKFCERILNPCELLPCLNNATCVSEQQNYKCRCMPGFTGKNCEEVIDYCKL
 Zebrafish    (360) KLNATCICVDPECLEQAEVCGTLPCLNGGICVVPNGQYHCRCRQGFSGKNCEEIIDFCKL
Drosophila      (1) --------------------------------------------------------MSNV
                                            EGF-like domain

     Human    (415) LSINCLNEEWCFNIIGRFKYVCIPGCTKNPCWFLKNVYLIHQHLCYCGVTFHGICQDKGP
   Macaque    (415) LSINCLNEEWCFNIIGRFKYVCIPGCTKNPFWFLKNVHLIHLHPCYYGITFHGICQDKGP
   Chicken    (414) LSINCLNEGLCLNIIGGFTCLCAPGWTGEFCQVAENACLIYPKSCSNGSTCIDMSQLGEQ
 Zebrafish    (420) LNINCLNEGLCLNRVGGYNCLCAPGWTGEFCQYLENACLAYPNRCLNGATCISMSQTTAP
Drosophila      (5) HQFDTQTMAESPQIRRDMGRLCATWPSKDSEDGAGTALRAATPLTANGATTTGLSVTLAP
                          EGF-CA domain
```

```
     Human   (475) AQFEYVWQLGFAGS-----------------------------------EGEKCQGVIDA
   Macaque   (475) AHFEYVWQLGFTGS-----------------------------------EGEKCQGVIDA
   Chicken   (474) PLFQCLCPHGFTGEFCKVQIDNCNSNLCENGGTCVNYEDHFKCICPMGFEGERCELDIDV
 Zebrafish   (480) PHYMCTCLPGYTGPYCEAEVNECDSSPCQHQGTCTDFVGYYKCTCPSGYTGIDCEIDINS
Drosophila    (65) KDMQRNHLLKMPTAT---------------------------------------------

     Human   (500) YFFLAANCTEDATYVNDPEDNNSSCWFPHEGTKEICANGCSCLSEEDSQEYRYLCFLRWA
   Macaque   (500) YFFLTANCTEDAIYVNNPEDNNSSCSFPCEGTKEICANGCSCLSEEDNQEYRYLCFLRWT
   Chicken   (534) CLFYNISCAPGAVCMNKSHGFNYTCLSPCIGKTEVCANGGSCFYDEGNQRSHCVCAHGWT
 Zebrafish   (540) CWLPNATCPPETLCVDLPGDQLFKCHTPCPHYLQPCANGGHCVLHN-ITSYSCVCAPGWT
Drosophila    (80) -----IEKPTITATIASS---------------SSTS------TSTTRKSVTATRSLKLN

     Human   (560) GNMYLENTTDDQENECQHEAVCKDEINRPRCSCSLSYIGRLCVVNVDYCLGNHSISVHGL
   Macaque   (560) SNMYLENITDDQENKSQHEAICEDEINRPRCSCSLSYIGRLCVVNVDYCLGNQSISVHGL
   Chicken   (594) GQTCLENINDCEINQCQHGATCEDEVNKYRCICPLGYTGTFCEIDIDNCIGN-QCSEYGF
 Zebrafish   (599) GATCLVNINECVQHRCQNRATCVDEVGGYSCLCGHGYTGVHCELDFCSGHQC---SEHAV
Drosophila   (114) PNILLPTLRILARGLLLPALILAILVG--SSQAGFACLSNPCVFGVCIDGLN---SSYSC
                                EGF domain

     Human   (620) CLALSHNCNCSGLQRYERNICEIDTEDCKSASCKNG------------------------
   Macaque   (620) CLALSHNCNCSDLQKYEGNICEIDIEDCKSVSCKNG------------------------
   Chicken   (653) CQDHLHNYSCICMLGYGGPFCEVEINECSSSPCKNGGICMNLIGSFSCHCAEGFKGETCT
 Zebrafish   (656) CVDQQHNYTCRCMLGYEGTLCELETDECKSAPCTNNATCIDLVAGYQCLCAPGFKGRTCS
Drosophila   (169) YCIDG----------YTGIQCQTNWDECWSSPCQNG------------------------

     Human   (656) ------------------------------------------------------------
   Macaque   (656) ------------------------------------------------------------
   Chicken   (713) AHVNECLDRPCWNGGTCEEDINGFKCNCPLGFEGLNCEINFDECTYGFCKNNSTCLDLIA
 Zebrafish   (716) ESMNECWSRPCNNGGSCIDLVNDYICNCPLGFTGHDCSMPATGCTSNPCNTKGTSMCEEQ
Drosophila   (195) ------------------------------------------------------------

     Human   (656) ------------------------------------------------------------
   Macaque   (656) ------------------------------------------------------------
   Chicken   (773) --DYSCVCPPGFTDKNCSTDIDECAFKPCQNGGHCHNLIGEFYCSCLPGFTGQFCEADVA
 Zebrafish   (776) QDGFKCVCHHGYTGLFCETSINHCVEGLCHHGSECVDLTKGFMCECLPGLRGRLCEVNID
Drosophila   (195) ------------------------------------------------------------

     Human   (656) ------------------------------------------------------------
   Macaque   (656) ------------------------------------------------------------
   Chicken   (831) ACLSQPCGASSICKDMSDGYVCFCAPGFIGNNCEIEVDECLSDPCHSGATCIDHLNGFSC
 Zebrafish   (836) DCLDKPCGALSICKDGINAYDCFCAPGFVGNNCEIEVNECLSQPCQNGASCSDELNSFSC
Drosophila   (195) ------------------------------------------------------------

     Human   (656) --------------------------TTSTHLRGYFFRKCVPGFKGTQCEIDIDECASHP
   Macaque   (656) --------------------------TTSIHLRGYFFYKCVPGFKGTRCEIDLDECALHP
   Chicken   (891) ICQGGFQGTTCETNINECHSSPCLHNATCADFVGGYECICLPGFTGTRCETDIDECASSP
 Zebrafish   (896) LCLAGTTGSLCEINIDECQSSPCMNNGTCLDLSDGFKCICPSGFSGPECSMDINECVSYP
Drosophila   (195) --------------------------GTCVDGVAYYNCTCPEGFSGSNCEENVDECMSNP

     Human   (690) CKNGATCIDQPGNYFCQCVPPFK-------------------------------------
   Macaque   (690) CKSGATCIDQPGNYFCQCGPPFK-------------------------------------
   Chicken   (951) CKNGATCIDQPGNYFCQCMAPFKGLNCEFRPCEASNPCENGAVCTEEMNLDAFPLGFQCQ
 Zebrafish   (956) CKNGGSCIDQPGNYYCRCLAPFKGLNCELLPCEAVNPCDNGAECVEEADLVLFPLGFQCR
Drosophila   (229) CQNGGLCRDR--------------------------------------------------
                          EGF-CA domain
```

```
     Human   (713) -----------------------------VVDGFSCLCNPGYVGIRCEQDIDDCILNACE
   Macaque   (713) -----------------------------VVDGFSCLCNPGYVGTRCEQNIDNCILNAFE
   Chicken  (1011) CVKGFAGPRCEINVNECSSNPCLHGYCYDIVNGFYCLCNPGYAGLTCEQDIDDCINNACE
 Zebrafish  (1016) CRKGFTGPRCEVNIDECSSNPCLNGFCYDAVDGFYCLCNPGYAGVRCEQHINDCASNMCE
Drosophila   (239) ------------------------------TNGYICTCQPGYLGSHCELDVAVCETGTGA

     Human   (744) ----HNSTCKDLHLSYQCVCLSDWEGNFCEQESNECKMNPCKNNSTCTDLYKSYRCECTS
   Macaque   (744) ----HNSTYKDLHLSYQCVCLSGWEGNFCEQESNECKMNPCKNNSTCTDLYKSYRCECTS
   Chicken  (1071) ----HNSTCVDLHLRYQCVCLPGWEGTFCEYESNECDSEPCRNNGTCTDLFNSYRCLCTA
 Zebrafish  (1076) ----NNSTCVDLHLSYNCLCLPGWEGEYCQRETNECLSNPCKNNATCTDLLNAYRCVCPQ
Drosophila   (269) RCQHGGECIEGPGLEFTCDCPAGWHGRICQEEINECASSPCQNGGVCVDKLAAYACACPM
                          EGF-CA domain                 EGF-CA domain

     Human   (800) GWTGQNCSEEINECDSDPCMNGGLCHESTIPGQFVCLCPPLYTGQFCHQRYNLCDLLHNP
   Macaque   (800) GWTGQNCSEEINECDSDPCMNGGLCHESTIPGQFVCLCPPLYTGQFCHQRYNPCDLLNNP
   Chicken  (1127) GWTGPDCSEDINECDSEPCLNGATCYESVKQGQFVCICPPFYTGDFCHQRFSPCELPYNP
 Zebrafish  (1132) GWTGLDCDEDVKECSSSPCLNGAHCVESDTPGEFSCTCPPFFTGPLCEQPYDPCELQRNP
Drosophila   (329) GYTGINCEEEILICADNPCQNNALCLMEEG--VPTCYCVPDYHGEKCEFQYDECQLG---
                                      EGF domain

     Human   (860) CRNNSTCLALVDANQHCICREEFEGKNCEIDVKDCLFLSCQDYGDCEDMVNNFRCICRPG
   Macaque   (860) CRNNSTCLALVDGNQHCICREEFEGKHCEIDVKECLFLSCQDYGDCEDMVNNFRCICRPG
   Chicken  (1187) CINNSTCLAQADGNPMCICKTGYEGTYCEVNSDECISHPCQNEGLCVDGINSYRCFCQHG
 Zebrafish  (1192) CLHNSTCRAQSDGTALCVCPVGFEGTRCEIDSDDCVSRPCQNRGICVDGVNSYSCFCEPG
Drosophila   (384) ----------------------------------PRCMNG---GVCIDGVDTFSCSCPPL
                         EGF domain                      EGF-domain

     Human   (920) FSGSLCEIEINECSSEPCKNNGTCVDLTNRFFCNCEPEYHGPFCELDVNKCKISPCLDEE
   Macaque   (920) FSGSLCEIEINECSSEPCKNNGTCVDLTNRFFCNCEPGYHGPFCELEVNKCKISPCLDEE
   Chicken  (1247) FTGTLCEVEINECLSRPCKNNGTCLDLINRFICNCAPGYYGSLCEIDINECETLPCLNGG
 Zebrafish  (1252) FSGLHCEEDINECASNPCQNQAVCQDLVNGFQCSCVPGYFGPHCNLDVNECDSSPCLHES
Drosophila   (407) LTGMLCECLMVGEESLDCNYTAPATQSPPRRTTTTSTMAPPTVRPVTPPETTVSPSR---
                                  EGF-CA domain

     Human   (980) NCVYRTDGYNCLCAPGYTGINCEINLDECLSEPCLHDGVCIDGINHYTCDCKSGFFGTHC
   Macaque   (980) NCVYRTDRYNCLCAPGYTGINCEINLDECLSEPCLHDGVCIDGINHYTCDCKSGFFGTHC
   Chicken  (1307) SCINRLGGYQCFCSPGFTGDRCETNTDECISTPCLNNGSCIDDINSYKCHCRSGFIGTNC
 Zebrafish  (1312) VCINKPGGFACVCSAGFSGKWCELNVDECKSNPCRNNGSCIDGLNGYQCVCSRGFMGDHC
Drosophila   (464) ---ASEEVEIIVVTTSAPAEVVTSVLSPSSSSSSSEEGVSVEIKTPTVAPPESGSHSISV
                        EGF domain                   EGF-CA domain

     Human  (1040) ETNANDCL-SNPCLHGRCTELINEYPCSCDADGTSTQCKIKINDCTSIPCMNEGF----C
   Macaque  (1040) ETNANDCL-SNPCLHGRCTEPINEYPCSCDADGTSIQCKIKINDCTSMPCMNEGF----C
   Chicken  (1367) ETNVNECW-PEPCLHGRCIDLIDGYQCSCEAGWTSSRCEININECESVPCINGGS----C
 Zebrafish  (1372) ERNTDECS-SGPCVHGSCLDEIDAFSCQCEVGWTGHRCQININECEAHPCLNGGS----C
Drosophila   (521) EQTTAVPAQPEPESEQEPESKPHPESESASESETETEEEIIPGTTARPPTSRSSSSSEES
                               EGF-like domain

     Human  (1095) QKSAHGFTCICPRGYTGAYCEKSIDNCAEPELNSVICLNGGICVDGPGHTFDCRCLPGFS
   Macaque  (1095) QKSAHGFTCICPRGYTGAYCEKSIDNCAEPELNSVICLNGGICVDGPGHTFDCRCLPGFS
   Chicken  (1422) QDLVNEFACICLTGYTGKECEFDIDICNEPTVSSVLCHNGGVCVDGPGRTFHCRCLAGFS
 Zebrafish  (1427) VDLLDKYACICADGFTGKNCDIDQNVCLQTSLNFSLCFNGGTCVDGPGVNFTCSCRPGFM
Drosophila   (581) PSIFTTLPPLPGKPQTSASSESSGEVVTSEEYTTVPHFEVSGSKSESG-SEEVTTVRPTA
                      EGF domain                        EGF domain

     Human  (1155) GQFCEININECSSSPCLHGADCEDHINGYVCKCQPGWSGHHCEN-ELECIPNSCVHELCM
   Macaque  (1155) GQFCEININECSSSPCLHGANCEDHINGYVCKCQPGWSGHHCEK-ELECVPNSCVHQLCM
   Chicken  (1482) GQFCEIEVNECNSSPCLHGSTCEDHVNGYTCQCQKGWEGLHCELDVDECISNPCIHGICV
 Zebrafish  (1487) GDFCEVEMNECCSEPCFNGAICQDLINGYQCHCRPGWTGLHCEDDINECLLQPCNQGMCI
Drosophila   (640) APSITISVDITSSGSSSSSSESVEVFTTPAPVFVQRVTTIETSISIDYVTPTPLPETTTP
                                 EGF-CA domain
```

```
      Human  (1214) ENEP--GSTCLCTPGFMTCSIGLLCGDEIRRITCLTPIFQRTDPISTQTYTIPPSETLVS
    Macaque  (1214) ENEP--GSTCLCTPGFMTCSIGLLCGDEIRRITCLTPSFQRTDPISTQTHTVPPSETLVS
    Chicken  (1542) QSDPSFGYSCFCKPGFVGRSCELNYNDCLIQSCSSGFLCVDGINNITCLPTISQS-----
  Zebrafish  (1547) QNEPGHGYTCFCRPGFVGENCEYNYDDCLIQSCPETFSCKDGINNVSCVPVKTDTSSLP-
 Drosophila   (700) RVVPVPRPTFAPEPPLDVVETTASTHHLWTEVPTTAAPFFTEYPAEVLITTHRTSAG---
                        EGF-like domain

      Human  (1272) SFPSIKATRIPAIMDTYPVDQGPKQTGIVKHDILPTTGLATLRISTPLESYLLQELIVTR
    Macaque  (1272) SFPSIKATRIPTIMDTYPVDQGPKQTGIVKHDILPTTGLATLRISTPLKSYLLEELIVTR
    Chicken  (1597) -----KKTEMAELFPTESLDN-----------------------------DLPSALAVSM
  Zebrafish  (1606) ----------------------------------------------PISVVSWRSTDIST
 Drosophila   (757) -----------------------------------------------RFTTVQPPAGVTT

      Human  (1332) ELSAKHSLLSSADVSSSRFLNFGIRDPAQIVQDKTSVSHMPIRTSAATLGFFFPDRRART
    Macaque  (1332) ELSAKHSLLSSTDVSSSPFLNFGIHDPAQIVQDKTSVSHMRIRTSAATLGFFFPDRRART
    Chicken  (1623) ELWSKHAIP---------------------------------------------------
  Zebrafish  (1620) ELQPTFAPVEN-------------------------------------------------
 Drosophila   (770) TSPTEDSSVELP------------------------------------------------

      Human  (1392) PFIMSSLMSDFIFPTQSLLFENCQTVALSATPTTSVIRSIPGADIELNRQSLLSRGFLLI
    Macaque  (1392) SFIRSSLMSDFIFPTQSLLFENYQTVASSATPTTSVIRSIPGADIELNRHSLLSRGFLLT
    Chicken  (1632) ------------------------------------------------------------
  Zebrafish  (1631) ------------------------------------------------------------
 Drosophila   (782) ------------------------------------------------------------

      Human  (1452) AASISATPVVSRGAQEDIEEYSADSLISRREHWRLLSPSMSPIFPAKVIISKQVTILNSS
    Macaque  (1452) AASISATPVVSRGAQEDIKEYSAVSLISRREHWRSLISSMSPIFPAKKIISKQVTILNSS
    Chicken  (1632) ------------------------------------------------------------
  Zebrafish  (1631) ------------------------------------------------------------
 Drosophila   (782) ------------------------------------------------------------

      Human  (1512) ALHRFSTKAFNPSEYQAITEASSNQRLTNIKSQAADSLRELSQTCATCSMTEIKSSREFS
    Macaque  (1512) ALHRFGTKAFIPSEYQAITEASSNQRLTNIKSQAADSLRELSQTCATCSMTEIKSSHEFS
    Chicken  (1632) ------------------------------------------------D---FHTEEVA
  Zebrafish  (1631) ----------------------------------------------------LQHTEQPA
 Drosophila   (782) ----------------------------------------------------TPHTPQIV

      Human  (1572) DQVLHSKQSHFYETFWMNSAILASWYALMGAQTITSGHSFSSATEITPSVAFTEVPSLFP
    Macaque  (1572) DQVLHSKQSHFYETFWMNSAILASWYALMGAQTITSGHSFSSATEITPSVAFTEVPSLFP
    Chicken  (1640) --QDFS------------------------------------------------------
  Zebrafish  (1639) DASFGG------------------------------------------------------
 Drosophila   (790) VTILDSN-----------------------------------------------------

      Human  (1632) SKKSAKRTILSSSLEESITLSSNLDVNLCLDKTCLSIVPSQTISSDLMNSDLTSKMTTDE
    Macaque  (1632) SKKSAKRTILSSSLEESITLSSNLDVNLCLHKTCLSIVPSQTISSDLMNSDLTSELTTDE
    Chicken  (1644) ------------------------------------------------------------
  Zebrafish  (1645) ------------------------------------------------------------
 Drosophila   (797) ------------------------------------------------------------

      Human  (1692) LSVSENILKLLKIRQYGITMGPTEVLNQESLLDMEKSKGSHTLFKLHPSDSSLDFELNLQ
    Macaque  (1692) LSVSENILKLLKIRQYGITTGPTEVLNQDSLLDMEKSKGSHTPFKLHPSDSSLDLELNLR
    Chicken  (1644) ------------------------------------------------------------
  Zebrafish  (1645) ------------------------------------------------------------
 Drosophila   (797) ------------------------------------------------------------
```

```
     Human  (1752) IYPDVTLKTYSEITHANDFKNNLPPLTGSVPDFSEVTTNVAFYTVSATPALSIQTSSSMS
   Macaque  (1752) SYPDVTLKTYSEITLANDLKNNLPPLTGSVPDFSEVTTNVAFYTVSATPALPIQTSSSMS
   Chicken  (1644) ------------------------------------------------------------
 Zebrafish  (1645) ------------------------------------------------------------
Drosophila   (797) ------------------------------------------------------------

     Human  (1812) VIRPDWPYFTDYMTSLKKEVKTSSEWSKWELQPSVQYQEFPTASRHLPFTRSLTLSSLES
   Macaque  (1812) VITPDWPYFIDYMTSLNKEVKTYSEWSKWELQPSVQYQEFPTASWHLPFTRSLTLSSLES
   Chicken  (1644) ------------------------------------------------------------
 Zebrafish  (1645) ------------------------------------------------------------
Drosophila   (797) ------------------------------------------------------------

     Human  (1872) ILAPQRLMISDFSCVRYYGDSYLEFQNVALNPQNNISLEFQTFSSYGLLLYVKQDSNLVD
   Macaque  (1872) IVAPQQLMISDFSCVCYYGDSYLEFQNVVLNPQNNISLEFQTFSSYGLLLYVKQDSNLVD
   Chicken  (1644) -YA------------RYYGDSYLEFQGLHLNVQNFIHLEFKTYKPDGLLLYIEESSETIG
 Zebrafish  (1645) ----------------YSGNSFLEFGGFEVAVPISVTVRFQTESMYGTLLYS--ASAKRS
Drosophila   (797) ---------EVIPSLITTTGSPTTHHHHHHHPHHEAEGTTLQPLEEDEHHHHHHHDEFTT
                                                       Laminin-G domain

     Human  (1932) GFFIQLFIENGTLKYHFYCPGEAKFKSINTTVRVDNGQKYTLLIRQELDPCNAELTILGR
   Macaque  (1932) GFFIQLSIENGTLKYHFYCPGEAKFKSINTAIRVDDGQKYTLLIRQELDPCKAELTILGR
   Chicken  (1691) QFLIQLFIRHGILQYQFVCGKATQVKNITTTARVDDGQWYKVQIRQNMKPCEAEMLILEV
 Zebrafish  (1687) VFFIKLYISNGILQYDFLCNQKQGVQRINTAQWVADGNEHVVIFRQCLFPCVAEVTVSGV
Drosophila   (848) PQPVEITTGHPLQTEDLIGVQEPAVVTTESPFAPAETTVVPVVVPATIAPLGTAAPPATP
                                      Laminin-G domain

     Human  (1992) NTQICESINHVLGKPLPKSGSVFIGGFPDLHGKIQMPVPVKNFTGCIEVIEINNWRSFIP
   Macaque  (1992) NTQTCESINHVLGKPLPKSGSVFIGGFPDLRGKIQMPVPVKNFTGCIEVIEINNWRSFIP
   Chicken  (1751) SAKTGIPSNFSSSPYGLETGSIFVGGLPYSSAIKQIPEPVYNFTGCIQVIEINNVGPFNF
 Zebrafish  (1747) RTVRSAPGNYTSALRLQRTDHVFIGGLPRHRSPYKEAEPFHNYTGCIEIIEINKLRRFHM
Drosophila   (908) APVPPATTTPPPSPPSLATETPTLPPTLPPVTLPPVTQPPPTIPPTPPSTQSAQTLPPPT
                                      Laminin-G domain

     Human  (2052) SKAVKNYHINNCRSQGFMLSPTASFVDASDVTQGVD---TMWTSVSPSVAAPSVCQQDVC
   Macaque  (2052) SKAVRNYHINNCRSQGLMLSPTASFVDASDVTQGVD---AMWTSVSPSVAAPSVCQEDVC
   Chicken  (1811) SNAVGRRNIDSCRTPVPTHLPTPFPTVSSDVLALPE---LVTPSLS-SPELPSVCQEGLC
 Zebrafish  (1807) DHAIARNNVDNCRSQWHHEPPTSSTHSPTLLITVETPP-GEWVRVLSPTQPAPVCPQGIC
Drosophila   (968) SAINVYTTPDGPPTASQTKPSVTESSEEVEGTNTVSTGGRGSGGVPEEKAGDVDCIKLGC

     Human  (2109) HNGGTCHAIFLSSGIVSFQCDCPLHFTGRFCEKDAGLFFPSFNGNSYLELPFLKFVLEKE
   Macaque  (2109) HNGGTCRPIFLSSGIVSFQCDCPLHFTGRFCEKDAGLFFPSFSGNSYLELPFLNFVLEKE
   Chicken  (1867) HNGGTCHPISLPTGAISFECDCQLHFTGRFCEKDVTLFIPSFNGNSYLELPSLTSVSQMR
 Zebrafish  (1866) LNGGTCRPVSLPSGASSFFCDCPLHFTGRLCEQDITVFSPRFDGNSFLELPSLTSLFQSD
Drosophila  (1028) YNGGTCVTTSEGS-----RCVCRFDRQGPLCELPIIIRNAAFSGDSYVSHRIYKDIGGHE
                            EGF domain

     Human  (2169) HN-------RTVTIYLTIKTNSLNGTILYSNGN-NCGKQFLHLFLVEGRPSVKYGCGNSQ
   Macaque  (2169) HN-------RTVTIYLTIKTNSLNGTILYSNGN-NFGKQFLHLFLVEGKPSVKYGCGNSQ
   Chicken  (1927) TASGQETS-NLTTLYLTVKTTAPSGTILYTSEK-NFGEQFLHLYLVEGRPTVRFSCGNSQ
 Zebrafish  (1926) TYFPSRSSEDKRILYLTMKSRTPHGSLLYCREQ-DLGERFLHVFLQNARAVARLGCG-AA
Drosophila  (1083) SLDAVLP----MHIQLKVRTRATNGLIMLAAAQGTKGGHYMALFLQKGLMQFQFSCGLQT
                                          Laminin-G domain

     Human  (2221) NILTVSAN-----YSINTNAFTPITIRYTTPVGSPGVVCMIEMTADGKPPVQKKDTEISH
   Macaque  (2221) NILTVSAN-----YSINTNAFTPITVRHTMPIGSPGVVCMIEMTADGKPPVQKKDTEISH
   Chicken  (1985) NILTVSGN-----QTISKGIFIPIIISYMLPVSSLEGYCMIEMAADRNPPVQHRLHLSYQ
 Zebrafish  (1984) HILTAVAA-----QNIRIDSLVAITVRYALPSQNNGQLCFIEIAADNGTANQQQKYMDEP
Drosophila  (1139) MLLSELETPVNTGHEITIRAELDFSRNYTHCNASLLVNDTLAMSGDQPTWLKLLPPRLHT
                                      Laminin-G domain
```

```
     Human  (2276) ASQAYFESMFLGHIPANVQIHKKAG----PVYGFRGCILDLQVNNKEFFIIDEARHGKNI
   Macaque  (2276) ASQVYFESMFLGHIPENVQIHKKAG----SVYGFRGCILDLQINNKEFFIIDEARRGKNI
   Chicken  (2040) ASQITFGSTFLGNVPVHKEVPECAG----QIRGYKGCIRDFQVNNKELFIIDDALGGRNV
 Zebrafish  (2039) VSEVVFGPTFLGGFPSVLELHHNSG----NVSGFIGCIRELQMGSKELYVVGEAIRGQNI
Drosophila  (1199) PEAILNTWLHLGGAPQAPIGLIIELPPAQSGSGFTGCLHTLRINGQAREIFGDALDGFGI
                                       Laminin-G domain

     Human  (2332) ENCHVPWCAHHLCRNNGTCISDNEN-----------LFCECPRLYSGKLCQFASCENNPC
   Macaque  (2332) ENCHVPWCAHHLCRNNGTCLSDSEN-----------LFCECPRLYSGKLCQFASCENNPC
   Chicken  (2096) ENCNVPICDYHPCRNGGTCTSDAEN-----------WFCECPKLYSGRLCQFMTCDESPC
 Zebrafish  (2095) QNCDAAVCQHQPCRNGGTCISDAES-----------WFCACPSLYSGKLCQFTACERNPC
Drosophila  (1259) TECGSLACLSSPCRNGAACIKIETNDLDENGEKAEKWKCKCPTGYMGPTCEISVCEDNPC
                                           EGF domain

     Human  (2381) GNGATCVPKSGTDIVCLCPYGRSGPLCTDAINITQPRFSGTDAFGYTSFLAYSRISDISF
   Macaque  (2381) GNGATCVPKSGTDIICLCPYGRSGPLCTDAINITQPRFSGTDAFGYTSFLAYSRISDISF
   Chicken  (2145) GNGATCFPKSRQDVVCLCPYGRSGILCNDVVNISQPSFSGTDVFGYTSFLAYSTIPDITF
 Zebrafish  (2144) ARGATCVPQTQLEAACLCPYGRQGLLCDEAINITRPKFSGLDEFGYSSYVAYPSIPSTGH
Drosophila  (1319) QYGGTCVQFPGSGYLCLCPLGKHGHYCEHNLEVALPSFSG-SVNGLSSFVAYTVPIPLEY
                          EGF domain

     Human  (2441) HYEFHLKFQLANNHSALQNNLIFFTGQKGHGLNGDDFLAVGLLNGSVVYSYNLGSGIASI
   Macaque  (2441) HYEFHLKFQLANNHSALQNNLIFFTGQKGHGLNGDDFLAVGLLNGSVVYSYNLGSGIASI
   Chicken  (2205) YYEFHLKFQLLNHHSALQDNLIFFTGQKGQGLNGDDFLVLGLCDGRVVYSYNLGSGTATI
 Zebrafish  (2204) FYEFHLKLTFANNASALRNNLILFSGQKGQGLSGDDFFALGVRNGRIVHKYNLGSGLATI
Drosophila  (1378) SLELSFKILPQTMS---QISLLAFFGQSGYHDEKSDHLAVSFIQGYIMLTWNLGAGPRRI
                                           Laminin-G domain

     Human  (2501) RSEP-----LNLSLGVHTVHLGKFFQEGWLKVDDHKNKSIIAPGRLVGLNVFSQFYVGGY
   Macaque  (2501) RSDP-----LNLSLGVHTVHLGKFFQEGWLKVDDHKNKSIIAPGRLVGLNVFSQFYVGGY
   Chicken  (2265) ISKP-----LDLTLNIHVIHLGRYLQKGWLKVDDQKNKTTTSPGRLVGLNVFSQFYLGGY
 Zebrafish  (2264) ISDR-----LNPRINIHTVHFGRYLKTGWLKVNGQKRRTGTSPGPLMGLNTFSQLYIGGY
Drosophila  (1435) FTQKPIDFRLDAPRVPYEIKVGRIGRQAWLSVDGKFNITGRSPGSGSRMDVLPILYLGGH
                                           Laminin-G domain

     Human  (2556) SEYTPDLLPNGADFKNGFQGCIFTLQVRTEKDGHFRGLGNPEGHPNAGRSVGQCHASPCS
   Macaque  (2556) SEYTPDLLPNGADFKNGFQGCIFTLQVRTEKDGHFRGLGNPEGHPNAGRSVGQCHASPCS
   Chicken  (2320) HEYTPELLPKGSRFKNGFQGCIFDVQVRTNMNQEFKSPGTPEGHPNSGRSVGQCKASPCS
 Zebrafish  (2319) EEYTPELLPPGSRFQNSFQGCIFDMLFRTRQDGKFHALGGPDIRPLSGRNVGQCGVNPCS
Drosophila  (1495) EIANFNTLPHDLPLHSGFQGCIYDVQLKAGQVTVP----LQETRGVRGRGVGQCGTRECH
                        Laminin-G domain

     Human  (2616) LMKCGNGGTCIESGTSVYCNCTTGWKGSFCTETVSTCDPEHDPPHHCSRGATCISLPHGY
   Macaque  (2616) LMKCGNGGTCIESGTSVYCNCTTRWKGAFCTETVSICDPEHDPPHHCSRGATCISLPHGY
   Chicken  (2380) LIKCRNGGKCMESGSTVYCNCLAGWKGAFCTEMVTVCDPEHDPPHLCKQGGTCVPLPNGY
 Zebrafish  (2379) LVFCHNGGTCVDSGSSVYCQCVFGWKGALCSEKVSFCDAEHIPPPFCARGSTCVPLSDGY
Drosophila  (1551) RHACQHDGACLQHGATFTCICQEGWYGPLCAQPTNPCDSFNN---KCYEDATCVPLVNGY
                          EGF domain                          EGF domain

     Human  (2676) TCFCPLGTTGIYCEQALSISDPSFRSNE--------------------------------
   Macaque  (2676) TCFCPLGTTGIYCEQALSISDPSFRSSE--------------------------------
   Chicken  (2440) MCHCPLGTSGTYCEQDISISDPSFRSNK--------------------------------
 Zebrafish  (2439) TCQCPLGSAGLHCQQAITISDPFFSGNQ--------------------------------
Drosophila  (1608) ECDCPVGRTGKNCEEVIRSLSDVSLTGRRSYLAVRWPYLYDGGDKLGAKRSQMVSYRNFT

     Human  (2704) -----------------------------------LSWMSFASFHVRKKTHIQLQFQPLA
   Macaque  (2704) -----------------------------------LSWMSFASFHVRKKTHIQLQFQPLA
   Chicken  (2468) -----------------------------------SSWMSFAPFYIRHKTHIKLQFQPLS
 Zebrafish  (2467) -----------------------------------SSWMSFPPINIRHRTHVQLQFQTLS
Drosophila  (1668) KKLMPPKPITTPSSHFVMKLLNEVEKQRSFSPVPLMGSKSFEEHHRVQFFFIEFQLRPLS
```

```
     Human  (2729) ADGILFYAAQHLKAQSGDFLCISLVNSSVQLRYNLG-DRTIILETLQKVTINGSTWHIIK
   Macaque  (2729) ADGILFYAAQHLKAQSGDFLCISLVNGSVQLRYNLG-DRTIILETLQKVTINGSTWHIIK
   Chicken  (2493) PDGILFYTAQRLGTQSGDFLCISLVNGFIQLRYNLG-DKTIVLQAVQKVHADGQTWHVLK
 Zebrafish  (2492) PEGILFYTAQHLSTHSGDFLSISLSAGFLQLRYNLG-NQTIVLQSPKELDVTGVRWHTVK
Drosophila  (1728) ERGLLLYFGTLNNNQDKKIGFVSLSLQGGVVEFRISGPSNHVTVVRSVRMIAIGEWHKIK
                                      Laminin-G domain

     Human  (2788) AGRVGAEGYLDLDGINVTEKASTKMSSLDTNTDFYIGGVSSLNLVNPMAIENEPVGFQGC
   Macaque  (2788) AGRVGAEGYLDLDGINVTEKASTKMSSLDTNTDFYIGGVSSLNLVNPMAIENEPVGFHGC
   Chicken  (2552) VGRVGNEGYVDLDGINITHTASAGMNVLDTHTDFYVGGVSSLNLVNSMATENEPTGFSGC
 Zebrafish  (2551) AGREGNSGFLIVDGESVTRNSSEGSTTLDVGANIFIGGISSLNTVSIDAVEKELVGFTGG
Drosophila  (1788) MAQRGRWLTLWVEGSASSALAPSAEVLVEPDSLLYIGGLKDVSKLPHNAISGFPIPFRGC
                                      Laminin-G domain

     Human  (2848) IRQVIINNQELQLTEFGAKGGSNVGDCDGTACGYNTCRNGGECTVN-GTTFSCRCLPDWA
   Macaque  (2848) IRQVIINYQELQLTEFGAKGGSNVGDCDGTACGYNTCRNGGECRVN-GTTFSCRCLPDWA
   Chicken  (2612) IREIVINDKELKLTVTDPKGGANIGDCDGTVCGYSVCKNNGTCQVE-SSGFSCSCPQGWI
 Zebrafish  (2611) IREVVVNGQELELTETGALDGANVGDWDGTACGYKVCKNGGHCHPSGDFSFTCICPSLWT
Drosophila  (1848) VRGLVVSGTRIVLNETNIVESRNIRDCDGTACGGDSCESGGHCWLDEKLQPHCICPEYAK
                                                       EGF domain

     Human  (2907) GNTCNQSVSCLNNLCLHQSLCIPDQSFS-YSCLCTLGWVGRYCENKTSFSTAKFMG-NSY
   Macaque  (2907) GNICNQSAYCLNNLCLHQSLCIPDQSFS-YSCLCTLGWVGRYCENKTSFTTAKFMG-NSY
   Chicken  (2671) GNTCEESVHCLHNRCRSQALCIPQPALLSYTCVCPLGWSGKHCDSKISFFTAKFVG-NSY
 Zebrafish  (2671) GSRCQQSIQCLNNLCQHNSVCIHNSTSASYSCMCSLGWTGTHCDREVTLKTIRFIG-NSY
Drosophila  (1908) GDRCEYSETCKLIPCKNNGRCLRSGRCS-----CPNGWGGFYCEIAMSKPTTPSFRGNSY
                                EGF domain

     Human  (2965) IKYIDPNYRMRN-----------LQFTTISLNFSTTKTEGLIVWMGIAQNEENDFLAIGL
   Macaque  (2965) IKYIDPNYRMRN-----------LQFTTISLNFSTTKTEGLIIWMGIAQNEENDFLAIGL
   Chicken  (2730) IKYIDPLYGKRD-----------LQYSRISLNFTTNQIEGLMVWLGKAEDEDNDFLAIGL
 Zebrafish  (2730) LKYKDPKYNSRN-----------LMHTEVSLNFSTSAGDGLIFWMGKAESEDDDHLAVGL
Drosophila  (1963) LILPPPRIPMKDKRRGPSLYVRPREAIQVSLNFSTIEPDGLLLWSEHERSK---FLGLGL
                                                Laminin-G domain

     Human  (3014) HNQTLKIAVNLGERISVPMSYNNGTFCCNK-WHHVVVIQNQTLIKAYINNSLILSEDIDP
   Macaque  (3014) HNQTLKIAVNLGERISVPMSYNNGTFCCNK-WHHVIVIQNQTLIKAYVNNSLILSEDIDP
   Chicken  (2779) ANGRLKVVINLGERISVPMIHSKDSICTDERWHFVTVIQNQTCIKVYLDEELIIFEDIDP
 Zebrafish  (2779) QDGYLKISVNLGERTALPLVYQNSFCCNYW--NYLSITHNRTLIQVYVNEERVIFEDIDP
Drosophila  (2020) EAGHLKLASNLLGSTNDTVRAPASGFIADGAWHWTSVLLDRSRLELQLDGEVIFTERLPE
                                      Laminin-G domain

     Human  (3073) --------------------HKNFVALNYDGICYLGGFEYGRKVNIVTQEIFKTNFVGKI
   Macaque  (3073) --------------------HKNFVALNYEGICYLGGFEYGRKVNIVTQEIFKTNFVGKI
   Chicken  (2839) --------------------HRKYTALNYGGICYFGGFEIGRKVHTATAGLFQKEFIGKI
 Zebrafish  (2837) --------------------FEQYVAVNYGGVIYLGGFELNRDVASVTSGVFTKGFEGSI
Drosophila  (2080) GGRSLGSTTPRSTLAGRRKNSSKEPTISYEDVFYLGGFPNSDSVSRRTKGREFDPFKGCL
                                      Laminin-G domain

     Human  (3113) KDVVFFQE-PKNIELIK-LEGYNVYDGDEQNEVT---
   Macaque  (3113) KDVVFFQD-PKKIELIK-LEGYNVYDGDEQNEVT---
   Chicken  (2879) KDIALFQD-SKKIQLMK-GEGYNVHNGDHRN------
 Zebrafish  (2877) KDVFLYQD-TKQLQFLQTCEGFNVYQGEE--------
Drosophila  (2140) QDIQFGAEPTAIISDFSTYQGENIGSCDLHGDEPLTV
```

Signal peptide | EGF domain | EGF-like domain

Laminin-G domain | Calcium-binding EGF-like domain

Residues identical in all sequences are white on a black background, whereas similar amino acids are white on a gray background. Residues that are present in at least three of the five proteins are indicated in black on a light gray background.
